# Microstructure‐Resolved Modeling to Predicting and Regulating Lithium Plating‐Stripping Dynamics on Graphite Electrodes

**DOI:** 10.1002/advs.202524109

**Published:** 2026-03-25

**Authors:** Heng Huang, Yang Li, Xinyu Liu, Zhifu Zhou, Wei‐Tao Wu, Lei Wei, Chengzhi Hu, Linsong Gao, Yubai Li, Yongchen Song

**Affiliations:** ^1^ Key Laboratory of Ocean Energy Utilization and Energy Conservation of Ministry of Education Dalian University of Technology Dalian China; ^2^ State Key Laboratory of Multiphase Flow in Power Engineering Xi'an Jiaotong University Xi'an China; ^3^ School of Mechanical Engineering Nanjing University of Science and Technology Nanjing China; ^4^ Department of Mechanical and Energy Engineering Southern University of Science and Technology Shenzhen China; ^5^ School of Mechanical Engineering and Mechanics Xiangtan University Xiangtan China

**Keywords:** graphite electrodes, lithium plating‐stripping reaction, microstructure‐resolved electrochemical model, structural regulation

## Abstract

The lithium plating reaction in graphite electrodes acts as a root cause for the accelerated degradation and the internal short circuits in lithium‐ion batteries. Here, an electrochemical model based on multi‐scale microstructural images was established to identify lithium plating‐stripping processes, thereby supporting the predictive outcomes of electrochemical monitoring techniques. Experiments revealed that the open‐circuit voltage differential curve (dOCV/dt) led to ambiguous delineation of the safe state‐of‐charge (SOC) operating range. The established lithium plating‐stripping model was used to compare with experimental results, revealing the dynamic evolution of electrode‐scale kinetics and quantified the impact of lithium metal residue on electrode performance. Ex situ X‐ray computed tomography (XCT) captured micrometer‐resolution microstructural details of graphite electrodes and plated lithium, enabling further correlation of spatially heterogeneous lithium plating‐stripping reactions with electrode microstructure. The sensitivity of lithium plating to electrode microstructure was examined at the particle scale, attributed to competition between electrode kinetic rates and active reaction areas. Theoretical mechanism analysis and experimental results from high‐energy‐density electrodes demonstrated that positioning small particles on the current collector side effectively mitigates solid‐state diffusion polarization while confining side reactions to a limited area. The integration of experiments and multiscale modeling elucidates the relationship between lithium plating‐stripping reactions and electrode structure, providing mechanistic insights for similar structural optimization designs.

## Graphical abstract

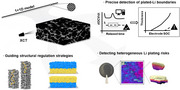

## 1 Introduction

The future of transportation electrification requires a combination of battery cost, range, and longevity. Currently, consumer concerns about charging time and range are major obstacles hindering the further development of the EV market [1]. At the negative electrode of lithium‐ion batteries (LIBs), a portion of the recyclable lithium cannot be embedded into the graphite (Gr) material before charging is complete. Consequently, it adheres to the electrode surface in the form of solid metal [2, 3, 4, 5]. Deposited lithium metal reacts with the electrolyte to form a solid‐electrolyte interphase (SEI) film, which consumes cycled lithium while simultaneously degrading ion transport [2, 5]. Although a high fraction of fresh lithium metal is reversible, a portion becomes inactive as it gets covered by the SEI film or detaches from the electrode surface, leading to rapid battery life degradation [6, 7]. This phenomenon frequently occurs under low‐temperature, high‐current, and overcharge conditions [8, 9]. Additionally, plated lithium has been found to lower the onset temperature of thermal runaway [10]. When lithium metal continuously grows in a dendritic form, it may cause a sudden drop in battery resistance, leading to either a soft short circuit (10^2^–10^3^ Ω) or a hard short circuit (< 1 Ω) [11]. Therefore, the key to preventing lithium plating‐related battery life degradation and ensuring operational safety depends on accurately predicting the onset of lithium plating and understanding the mechanisms governing its progression.

Post‐mortem characterization techniques provide direct evidence for the occurrence of lithium plating reactions, including scanning electron microscopy (SEM), optical microscopy [3, 4], mass spectrometry titration [12], X‐ray/neutron diffraction [5, 13], and nuclear magnetic resonance (NMR) [12, 13]. Although these techniques can be integrated into in situ technologies by correlating with battery operation processes, the customization involving specific battery structures enables broader application of certain in situ electrochemical techniques [14], such as impedance spectroscopy (EIS) analysis [15, 16] and relaxation open‐circuit voltage (OCV)/differential open‐circuit voltage (dOCV/dt) [7, 17]. Among these, identifying the voltage plateau characteristics after battery relaxation has emerged as one of the most promising in situ techniques for detecting lithium plating. When lithium metal coexists with lithiated graphite products (LiC_6_/stage I), the interphase rebalancing induces a mixed‐voltage plateau (0–0.085 V) in the voltage trace. This plateau corresponds to the peak signal in the dOCV/dt curve [12, 17, 18]. Although this technique serves as an effective indicator for plated lithium, it can only detect the reversible portion, and its detection capability depends on the amount of plated lithium [7, 17]. At high C‐rates, the inability to detect trace plated lithium at an early stage precipitates accelerated irreversible‐capacity fade and heightened safety hazards in later cycles [19, 20]. For small amounts of plated lithium, high‐precision coulombic efficiency (CE) can detect minute capacity decay (0.01%) caused by irreversible lithium metal [21], because plating/stripping is far less reversible than intercalation/de‐intercalation. Therefore, until in‐operando or in situ techniques with higher sensitivity emerge, combining complementary electrochemical methods with post‐mortem characterization remains the most reliable route to refine the detection of plating onset and to quantify the amount of plated metallic lithium. Nevertheless, none of these techniques can correlate the detection conditions with the electrode structure to provide a physical understanding of the onset and evolution of lithium plating. Newman et al. [22] developed a numerical framework based on the 1+1D (pseudo‐two‐dimensional, P2D) model of electrode physical processes, elucidating the mechanism evolution of lithium plating‐stripping reactions under homogeneity assumptions. By incorporating experimentally calibrated parameters, this model significantly enhances the quantitative accuracy of lithium plating capacity.

Unfortunately, homogeneous geometric modeling struggles to identify heterogeneous structural details within graphite electrodes (such as particle size, sphericity, and pore distribution) [23], thereby ignoring the distributed heterogeneity of state of charge (SOC) and reactions across multiple scales [24]. Parmananda et al. [25] demonstrated through analysis of 14 commercial graphite electrodes that structural heterogeneity at both the electrode and particle scales results in significant differences in lithium plating performance. Lu et al. [3] observed a dependence of particle‐level kinetic inhomogeneity on structural morphology (orientation, sphericity, etc.) through a phase‐field model combined with in situ optical microscopy and microstructure analysis, thereby initiating early electroplating. Additionally, lithium metal undergoes oxidation reactions at high potentials and re‐intercalates into graphite materials. Although the stripping process does not induce direct loss of electrode capacity, the lithium metal stripping mechanism is influenced by structural heterogeneity, leading to non‐uniform spatial distribution of inactive lithium [26]. Pan et al. [27] observed the lithium plating‐stripping process using in situ synchrotron X‐ray computed tomography (XCT) and found that the uniformity of the plated lithium layer is governed by the microstructure of porous graphite. Also, the stripping process may cause lithium metal to detach from the substrate, leading to local pore blockage and depletion of lithiation sites [28]. Therefore, models incorporating microstructural analysis become essential to elucidate these heterogeneous degradation phenomena. Although microstructural geometric modeling tools have been employed in several studies [3, 29, 30], most works focus exclusively on the lithium plating process while simply neglecting the stripping reaction, and the lack of validation between lithium plating models and experimental results may lead to significant errors in quantitative predictions of battery lifespan degradation. Additionally, the low X‐ray attenuation coefficient of graphite electrode materials poses substantial challenges for CT image segmentation. Consequently, virtual microstructure generation has become a prevalent approach in such studies. In graphite electrodes exhibiting substantial heterogeneity at the particle scale, interfacial side reactions during lithium plating are strongly dependent on the spatial morphology of particles. Thus, models based on the actual microstructures of graphite electrodes remain fundamental to achieving high‐fidelity performance predictions.

Electrolyte modification [31] and charge/discharge protocol design (such as multi‐stage charging, pulse charging protocols, etc. [18]) have been demonstrated to improve electrode interface reaction kinetics or overpotentials, thereby preventing lithium plating or enhancing its reversibility. Besides, due to the longer solid‐state diffusion (SSD) path, larger Gr particles exhibit early saturation at the surface, inducing the early onset of lithium plating [4]. Although smaller particles have shorter transport paths, their larger specific surface area tends to cause particle agglomeration during slurry preparation, as well as accelerate parasitic reactions during subsequent electrochemical cycles [32, 33]. For electrodes with higher energy density, longer liquid‐state diffusion (LSD) distances, and higher active material (AM) fractions make electrode porosity invaluable. However, experiments have observed that smaller particle sizes often result in a more tortuous and dispersed pore distribution [34], thereby impairing mass transport within the electrolyte. The influence of particle size distribution (PSD) and spatial arrangement of graphite materials on electrode macroscopic performance involves physical characteristics across multiple scales. Modeling based on microstructure facilitates a thorough understanding of this influence mechanism.

This work established an electrochemical model for lithium plating‐stripping kinetics based on electrode microstructure images from different dimensions (2D‐SEM or 3D‐XCT). Experimental curves were used to calibrate the diffusion‐reaction kinetic parameters underlying the model and to validate the results of battery life loss caused by lithium plating, thereby enabling prediction accuracy to effectively compensate for detection errors in electrochemical differential voltage techniques. Advanced machine learning methods were employed to obtain the two‐phase structure of XCT images from graphite electrodes, enabling this 3D microstructural model to reveal the correlation between electrode structural heterogeneity and the lithium plating‐stripping process. The plated lithium metal on cycled electrode samples was ex situ reconstructed, revealing a distinct analysis of its non‐uniform 3D spatial distribution. The impact of this distribution on electrode mass diffusion and the kinetics of electrochemical side reactions was modeled and analyzed. Finally, the impact of particle size and position regulation on lithium plating and rate performance was investigated. Meanwhile, this study provided a priori theoretical guidance for optimizing the experimental approach to delaying lithium plating through structural modification.

## 2 Results and Discussions

### 2.1 Monitoring Lithium Plating in Graphite Electrodes Through Differential Voltage Curve Experiments

Differential open‐circuit voltage curves are a widely adopted method for online monitoring of lithium plating onset [17, 18]. First, Li||Gr half‐cells with a mass loading of 2.16 mAh/cm^2^ were assembled. After three formation cycles (see Method 4.2), the cells underwent the discharge‐relaxation cycle shown in Figure S1 to determine the onset SOC for lithium plating. Graphite electrodes were lithiated to different SOCs and then relaxed for 30 min. During relaxation, deposited lithium exhibits enhanced oxidizing properties, driving lithium ions to re‐intercalate into graphite particles. This induces an additional plateau in the voltage curve (Figure S2). Additionally, at higher SOC levels, solid‐state diffusion within the electrode relaxes the interfacial lithium concentration. This relatively slow process is identified as the second voltage plateau. Unique material structures, such as larger particles, experienced spontaneous phase equilibrium after current interruption [3], which also contributed to the emergence of the second voltage plateau.

**FIGURE 1:**
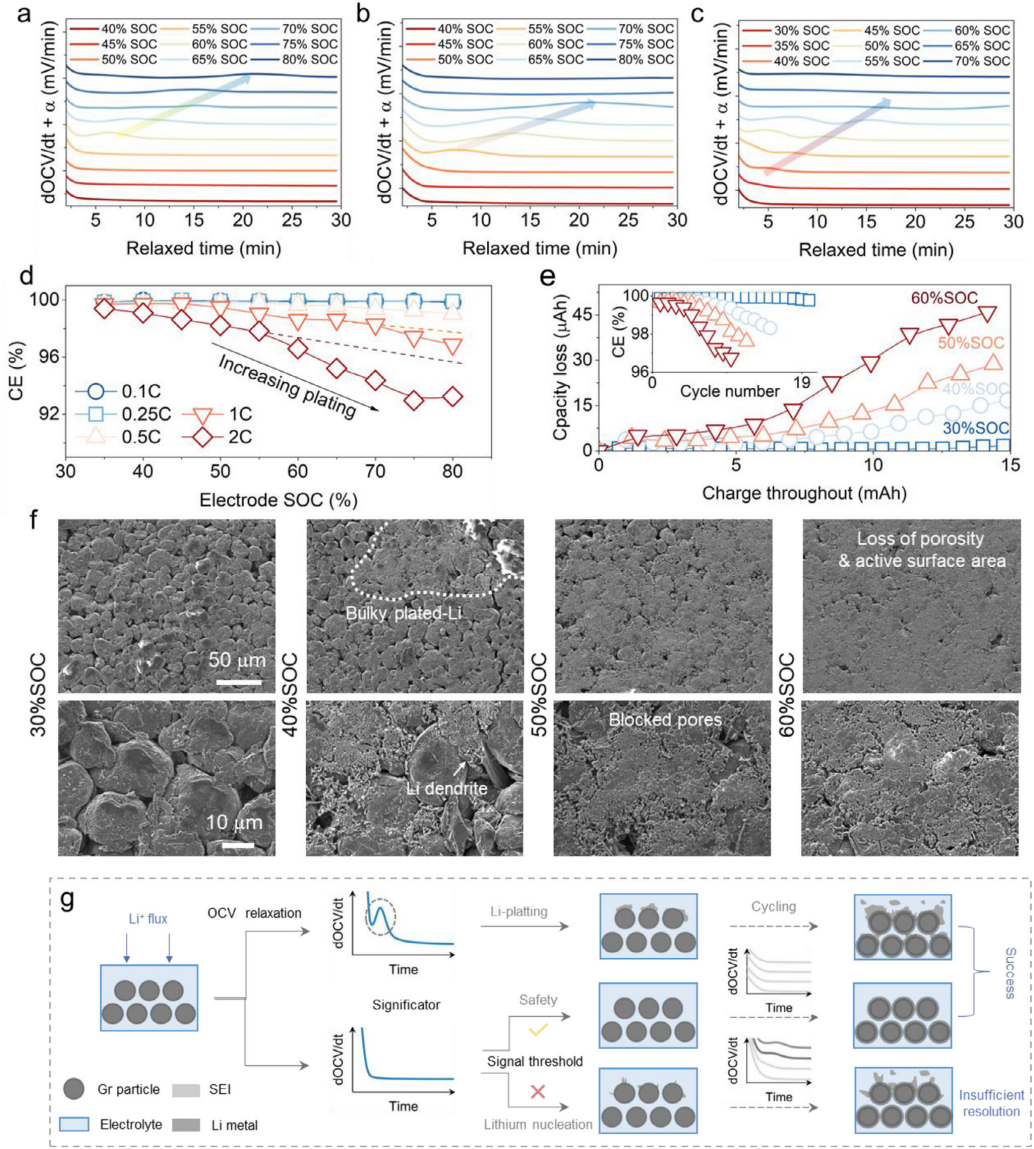
Online monitoring methods for lithium plating. (a–c) Differential relaxation voltage curves of the Li||Gr half‐cell after discharge: (a) 0.5C, (b) 1C, (c) 2C. (d) Variation of battery coulombic efficiency (CE) with SOC at different discharge rates. (e) Capacity loss during cycling at different discharge SOC under 1C rate. (f) SEM images of the Gr electrode surface after different SOC cycles. (g) Schematic diagram illustrating differential voltage curve monitoring for different scenarios in lithium plating.

Note that half‐cell discharge corresponds to full‐cell charging, where the graphite electrode experiences lithiation/potential reduction. At various rates, peaks began to appear in the battery's dOCV/dt curve as SOC increases, persisting throughout subsequent discharge cycles (Figure 1a–c). Consequently, the SOC corresponding to the earliest peak was typically adopted as the battery's safety boundary. Note also that the onset SOC of peak occurrence began to decrease with increasing discharge rate (60% SOC at 0.5C, 50% SOC at 1C, and 40% SOC at 2C). A potential reason was that the discharge current increased diffusion and reaction polarization within the electrode, favoring the thermodynamics of lithium plating.

The occurrence of a peak is regarded as the moment at which all reversible lithium has been fully stripped. The stripping time required for reversible lithium exhibits an increase with the SOC and discharge rate, representing the increased amount of deposited lithium. However, at higher rates and SOCs, substantial plated lithium could not be fully stripped within 30 min (Figure 1b,c). Most of the remaining reversible lithium was further stripped during subsequent charge–discharge cycles. The fact is that plated lithium may get covered by the SEI film or become disconnected from the substrate, thereby transforming into dead lithium [28], which can be corroborated by the battery's coulombic efficiency (CE) (Figure 1d). At 0.5C, all reversible lithium could be completely stripped during relaxation, so the CE exhibited a linear relationship with SOC. However, when the discharge rate increased to 1C or 2C, the irreversible reversible lithium caused the CE to deviate from the linear relationship and drop sharply.

Limited by the weak reaction current and the high voltage signal detection sensitivity, the lithium plating onset SOC determined by this method has recently come under increasing scrutiny [35]. To verify this hypothesis, for the determined 65% lithium plating onset SOC at 1C (Figure 1b), we assembled four additional cells to perform discharge‐relaxation cycles at the specified 30%–60% SOC range. Cells cycled at a 1C rate with consistent charge for all four SOCs to eliminate the influence of different SOC conditions on Li deposition. Except for 30% SOC, cycles below 65% SOC experienced significant capacity loss and CE decline (Figure 1e).

Close inspection of post‐mortem SEM images (Figure 1f) revealed that, even though the cell SOC never reached the detection onset threshold of 65%, the electrode surface was already covered with extensive plated lithium. At higher SOC levels (> 40%), l the accumulation of lithium dendrites causes the deposits of bulk lithium, clogging electrode pores and active surfaces. Cross‐sectional and magnified views in Figure S3b–d clearly illustrated this phenomenon. Additionally, batteries cycled at 30% SOC exhibited minimal lithium capacity loss prior to termination, with no signs of plated lithium observed in SEM images. The slight capacity loss is generally attributed to minor tearing and regeneration of the SEI layer caused by graphite particle expansion [36]. Therefore, the lithium plating onset SOC determined by online monitoring methods did not guarantee the absence of lithium plating at lower capacities (Figure 1g). Under conditions where no differential voltage peak signal is detected, fortunate batteries may successfully avoid lithium plating during cycling (30% SOC). However, cycling at 40% SOC indicates that even the absence of peak characteristics cannot guarantee the absence of lithium plating reactions during cycling. Consequently, avoiding lithium plating urgently requires the development of more sensitive and faster monitoring tools.

### 2.2 1+1D Electrochemical Model for Predicting Lithium Plating‐Stripping Process

#### 2.2.1 The Development of a Lithium Plating‐Stripping Model

The 1+1D electrochemical model based on electrode physical parameters enables rapid, real‐time, and accurate prediction of internal battery kinetics. Based on this model framework (detailed equations and descriptions are presented in Supplementary Method 1), the coupling relationship between lithium intercalation and lithium plating/stripping reactions is established by analyzing events at different time points at the graphite electrode interface, thereby constructing the complete physical process, including side reactions during cycling.

**FIGURE 2:**
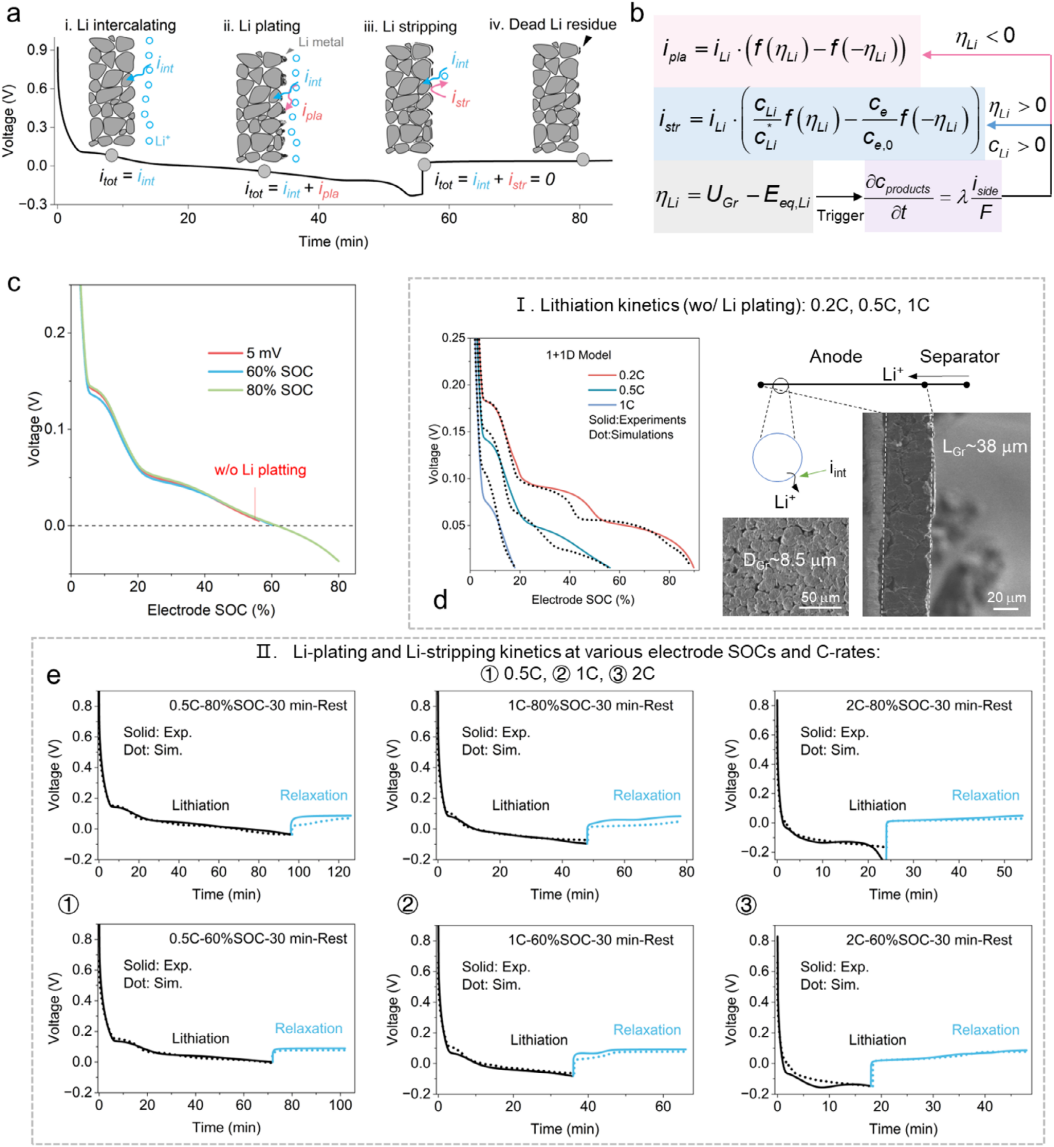
Establishment and experimental validation of a 1+1D model considering lithium plating‐stripping kinetics. (a) Cell voltage curves incorporating lithium plating‐stripping. (b) Coupling mechanism of lithium plating‐stripping reactions determined based on individual physical processes. (c) Trace of graphite electrode voltage curves under different SOC conditions. (d) Physical parameter calibration of the model and experimental validation of electrochemical rate curves. (e) Voltage curves at different SOCs and discharge rates were employed to validate the lithium plating‐stripping model.

Figure 2a displays a typical voltage curve of a Li||Gr half‐cell exhibiting lithium plating‐stripping behavior, and the reactions occurring within the cell at various time points were also shown in the inset. In the model, the triggering mechanism for each reaction was primarily determined by the overpotential η_Li_ (η_Li_ = U_Gr_—U_eq,Li_) relative to lithium metal, as shown in Figure 2b. Although recent studies had experimentally determined lithium plating potentials significantly below 0 V [3, 8], the widely adopted 0 V was used here as the general trigger condition for lithium plating‐stripping, accounting for variations in operating conditions and electrode materials [37]. Consequently, the reaction processes in each stage depicted in Figure 2a could be analyzed as follows.

During the initial discharge phase, conditions for lithium metal deposition have not yet been met (η_Li_ < 0). Only lithium ion (Li^+^) diffusion and reaction in graphite particles occur. That is, the internal current density (i_tot_) is governed by the charge transfer reaction at the particle surface (i_int_):

(1) $$\left\{\begin{array}{c} i_{tot}=i_{int}\\ i_{int}=\alpha_{s}i_{0}\times \left[exp\left(\frac{\alpha \eta_{ct}F}{RT}\right)-exp\left(-\frac{\left(1-\alpha \right)\eta_{ct}F}{RT}\right)\right] \end{array}\right.;\;\eta_{Li}>0$$

where i_0_ is the interfacial exchange current density, α_s_ is the specific surface area of the electrode, α is the charge transfer coefficient, η_ct_ is the overpotential for charge transfer reactions on the graphite particle surface, R is the universal gas constant, F is Faraday's constant, and T is the operating temperature.

i. As the cell continues discharging, increased mass transfer and reaction resistance within the graphite electrode cause its overpotential relative to metallic lithium to decrease below 0 V. At this point, a portion of Li^+^ is plated (Figure 2a), and the total current density (i_tot_) is governed by two parallel electrochemical reactions, namely the insertion reaction current (i_int_) and the lithium plating reaction current (i_pla_):
(2) $$\left\{\begin{array}{c} i_{tot}=i_{int}+i_{pla}\\ i_{pla}=\alpha_{s}i_{Li}\times \left[exp\left(\frac{\alpha_{Li}\eta_{Li}F}{RT}\right)-exp\left(-\frac{\left(1-\alpha_{Li}\right)\eta_{Li}F}{RT}\right)\right] \end{array}\right.;\;\eta_{Li}<0$$

i_Li_ is the current density constant for the lithium plating reaction, and α_Li_ is the charge transfer constant for the lithium plating reaction. The amount of metallic lithium generated by the lithium plating reaction can be estimated based on the law of conservation of mass [38]:

(3) $$\frac{\partial c_{Li}}{\partial t}=-\alpha_{s}\cdot \frac{i_{pla}}{F};\;\eta_{Li}<0$$

c_Li_ denotes the concentration of metallic lithium.

ii. The current interrupts at the end of cell discharge, transitioning into the voltage relaxation phase. During this process, a part of the metallic lithium inside the electrode strips off due to its electrochemical reactivity, which is known as reversible lithium plating. The stripping reaction of metallic lithium is influenced by the total amount of plated lithium and the liquid‐phase ion concentration, which can be described using the redox concentration‐dependent Butler‐Volmer equation [38, 39]. Simultaneously, the stripped lithium reinserts into the graphite electrode. This implies that the electrode current density at this stage is governed by the stripping current density (i_str_) and the charge reaction current density (i_int_):
(4) $$\begin{array}{ccc} & & \left\{\begin{array}{c} i_{tot}=i_{int}+i_{str}\\ i_{str}=\alpha_{s}i_{Li}\times \left[\frac{c_{Li}}{{c_{Li}}^{*}}exp\left(\frac{\alpha_{Li}\eta_{Li}F}{RT}\right)-\frac{c_{e}}{c_{e,0}}exp\left(-\frac{\left(1-\alpha_{Li}\right)\eta_{Li}F}{RT}\right)\right] \end{array}\right.;\\ & & \;\eta_{Li}>0,c_{Li}>0 \end{array}$$

where c_Li_^*^ denotes the experimentally calibrated reference metallic lithium concentration, and c_e,0_ represents the reference electrolyte salt concentration. Note that the lithium stripping reaction at this stage requires the presence of metallic lithium concentration, thus preventing stripping from occurring during the first discharge cycle. To predict the reversible portion of lithium plating (c_Li,rev_), this is quantified by assuming a reversible coefficient (λ) [39, 40]:

(5) $$\frac{\partial c_{Li,rev}}{\partial t}=\alpha_{s}\cdot \lambda \frac{i_{str}}{F};\;\eta_{Li}>0,c_{Li}>0$$

through calibration of experimentally measured relaxation curves, the reversible coefficient was determined to be 0.4 (Figure S18).

iii. After a long relaxation period, the reversible portion of the plated lithium is completely detached and embedded into the graphite electrode. However, a portion of lithium remains trapped on the electrode surface and cannot be stripped off (Figure 1f), directly causing the loss of cell CE, which is referred to as dead lithium. Although dead lithium can only be determined after the stripping reaction concludes, the model can predict the real‐time quantity of dead lithium (c_Li,dead_) during cell operation based on the total amount of plated lithium or by employing a reversible coefficient:
(6) $$\left\{\begin{array}{c} \frac{\partial c_{Li,dead}}{\partial t}=\alpha_{s}\cdot \left(1-\lambda \right)\frac{i_{str}}{F}\\ or,\\ c_{Li,dead}=c_{Li}-c_{Li,rev} \end{array}\right.;\;\eta_{Li}>0,c_{Li}>0$$

Please note that the work focuses on predicting lithium plating‐stripping kinetics during a single discharge relaxation process. The relatively low lithium plating amount and short operating time support the assumption of minimal secondary SEI film formation [38, 41]. Moreover, all cells used for model calibration underwent a pre‐formation step involving three constant current constant voltage protocols (Method 4.2), which is considered to establish a robust SEI film. Therefore, real‐time monitoring of the SEI film and its evolution is not considered here.

The reaction switching mechanism for lithium plating‐stripping described above was summarized in Figure 2b and coupled into an electrochemical model, sensed by key electrode physical parameters. This model was then used to predict lithium plating‐stripping behavior during the operation of the Li||Gr half‐cell. The model primarily determined information related to electrode structure, such as the dimensions of the graphite anode (particle size, thickness), as shown in Figure 2d, and the heterogeneous microstructure discussed in subsequent sections (detailed model equations are included in the Supplementary Methods). The calibration process for additional lithium plating‐stripping model parameters was shown in the Supplementary Methods.

Based on the overlapping voltage curve trajectories at different SOCs (Figure 2c) and Equations (1) and (2), only intercalation reactions occurred in the electrochemical system prior to lithium plating. Once lithium plating initiates, the new lithium plating current density competes with the lithium intercalation reaction. This implies that, using the onset of lithium deposition as a reference, both the lithium intercalation reaction process and the multi‐reaction process can be independently verified. First, the validation of lithiation kinetics at different discharge rates demonstrated high consistency between the model and experimentally measured curves in terms of capacity and decay trends (Figure 2d). Regrettably, as reported in the works of Lu et al. and Gao et al. [3, 4], the unique phase transition plateau of graphite as a phase‐change material at a specified SOC could not be predicted by the solid solution model employed in this work. Undeniably, the voltage curves at different rates confirmed the model's predictive capability regarding changes in the internal kinetic processes of the electrode. A voltage curve under different discharge rates and SOCs was used to validate the model's predictive capability for lithium plating‐stripping kinetics in batteries (Figure 2e). The model achieved good prediction results across various discharge rates and SOCs, particularly during the lithiation/lithium plating phase. The open‐circuit voltage predicted by the model for the stripping process is slightly lower than the experimental curve, which may be attributed to the simplified particle structure heterogeneity in the 1+1D model, leading to variations in the interfacial lithium content of the electrode. Although the solid solution model cannot predict the true concentration gradient within graphite particles, numerous studies indicate that the solid solution model possesses the capability to predict lithium plating risks as effectively as phase separation models (PSM) [42, 43]. Furthermore, additional validation of the model‐predicted capacity loss using coulombic efficiency losses (CIEs) under different operating conditions (Figure S4) yields consistent results, further demonstrating the model's excellent predictive accuracy.

#### 2.2.2 Lithium Plating‐Stripping Behavior During Cell Operation

**FIGURE 3:**
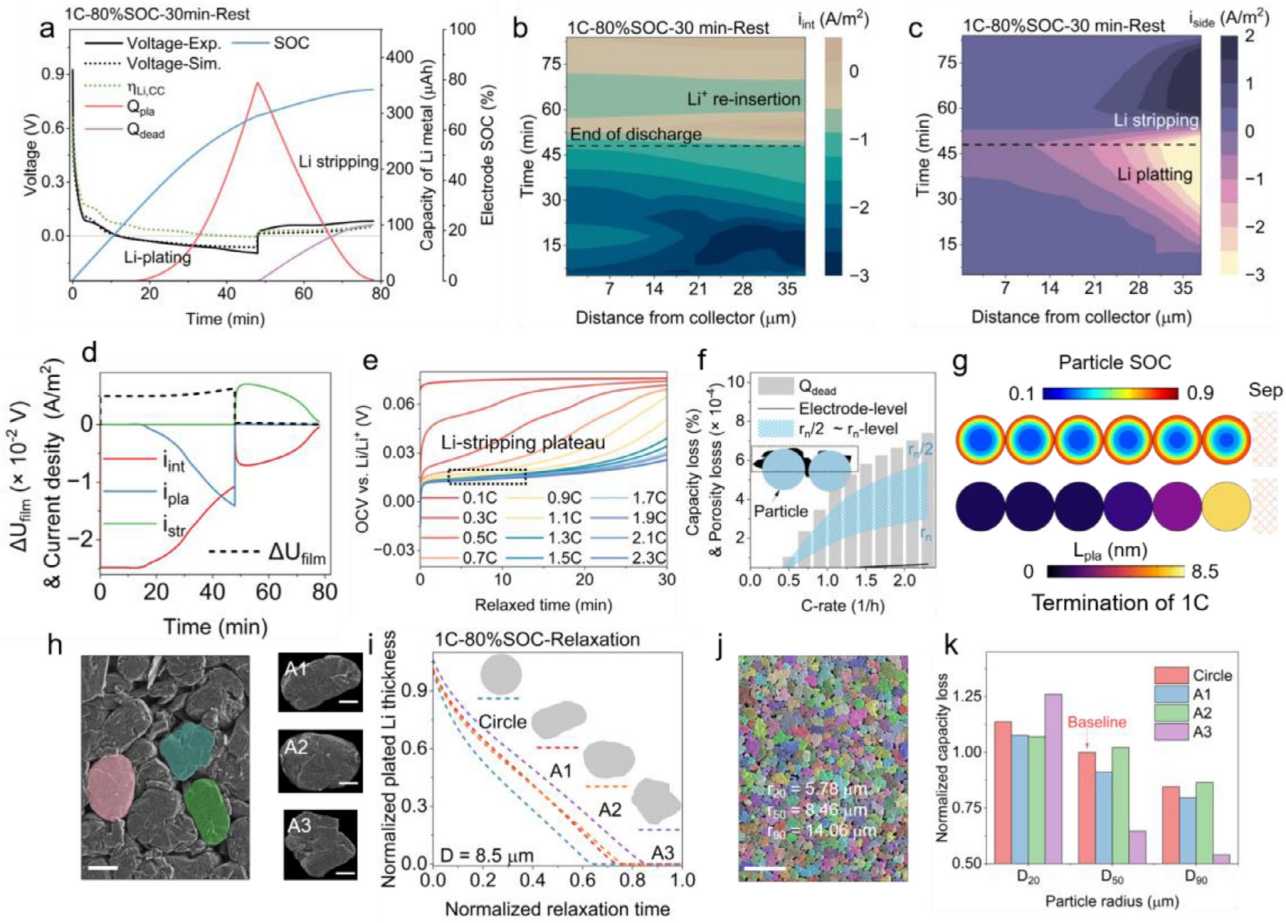
A 1+1D model investigates the lithium plating‐stripping mechanism. (a) Relationship between cell voltage, SOC, reaction products, and overpotential vs. time at a 1C rate. (b,c) Spatiotemporal evolution of electrode reaction current densities: (b) Insertion/extraction current density i_int_. (c) Lithium plating/stripping current density i_pla_/i_str_. (d) Evolution of average electrode current density and the film potential. (e) Cell voltage curves during relaxation periods at different discharge rates to 80% SOC. (f) Predicted battery capacity loss and electrode porosity loss at various rates. (g) Particle SOC and deposited lithium thickness distribution at the end of 1C discharge. (h–k) Particle morphology‐dependent lithium plating‐stripping kinetics: (h) Particles extracted from SEM images. (i) Effect of particle morphology on plated lithium thickness. Results for various particle morphologies normalized based on their initial lithium plating amounts. (j) SEM image‐dependent particle segmentation. (k) Influence of particle size and morphology on electrode capacity loss.

Voltage and lithium plating‐stripping kinetics were correlated with the lithiation‐relaxation process at a 1C discharge rate (Figure 3a). The plated lithium capacity (Q_pla_) in the electrode began to increase when the overpotential at the local position was less than 0 V, and then started to decrease after current interruption with the occurrence of stripping reaction. Due to the tortuous Li^+^ transport pathways within the electrode, significant potential differences existed between the separator and current collector (η_Li,cc_). During the relaxation period, irreversible capacity loss (Q_dead_) also increased, resulting in approximately 100 µAh of dead lithium at the end of the cycle and leaving 2.5 µAh of reversible lithium not stripped in time. Note that the reversible lithium stripped from the graphite particles would be reinserted into them, manifesting as a gradual increase in SOC. This phenomenon could be more intuitively observed through the spatiotemporal evolution of current density as reflected in electrode thickness (Figure 3b,c). During the relaxation phase, the stripping current i_str_, like other reaction currents, was concentrated near the separator (Figure 3c), while Li^+^ re‐insertion occurred throughout the entire electrode thickness (Figure 3b). Graphite particles near the separator exhibited higher interfacial lithium concentrations, lower solid‐phase diffusion rates as well as salt concentration and potential gradients across the electrode thickness, which promoted the diffusion of exfoliated Li^+^ toward the current collector side. This was consistent with reports by Ringler et al. [40] and Yang et al. [38]. Another key finding was that the emergence of lithium plating at the separator weakened the strength of the intercalation reaction (compared Figure 3b,c), and the lithium plating current overtook the intercalation current before discharge completion (Figure 3d). This competitive effect slowed the rate of capacity or SOC increase in graphite (Figure 3a). Simultaneously, the increased lithium plating current implied thicker plated lithium layers, thereby elevating the film overpotential during discharge (Figure 3d).

#### 2.2.3 Hazards of Lithium Plating to Cell Capacity and Structural Parameters

Compared to experimental methods, the model possessed the capability to predict extremely small amounts of plated lithium and could determine the approximate location of lithium distribution on the electrode. For cells discharged to 80% SOC, the relaxation voltage plateau of lithium stripping gradually extends as the discharge rate increases (Figure 3e), and the capacity loss caused by dead lithium also increased accordingly (Figure 3f). The capacity loss increased by 6% with the discharge rate increasing from 0.5C to 2.1C. Additionally, dead lithium occupied the pore space within the electrode. At 2.1C, charging to 80% SOC resulted in a loss of approximately 0.6% in electrode porosity. Note that the generated dead lithium is typically distributed on the surface of electrode particles (Figure 3g), and the prediction was confirmed by surface and cross‐sectional SEM images (Figure 1F; Figure S3). We attributed this primarily to the liquid‐state diffusion (LSD) limitations, as evidenced by the gradient distribution of particle SOC across thickness (Figure 3g). Remarkably, we defined the SOC value at the electrode level as a percentage, while the SOC value within the particle level was defined as a decimal. With the homogeneous spherical particles defined in the 1+1D model, assuming the deposited metallic lithium was distributed on the surface of the first layer of particles, the local porosity loss would further increase to between 2.8% (within the volume range of r_n_ from the first layer of particles) and 5.7% (within the volume range of r_n_/2 from the first layer of particles). Yang et al. [44] investigated the porosity loss of the full cell during cycling. After 2500 cycles, the local porosity near the separator began to drop sharply, a phenomenon that also corresponded to the distribution of plated lithium (Figure 3g). Beyond this, the hindering effect of the SEI film on ion transport was also critically important in practice and has attracted significant research interest. Deposited metallic lithium reacted with the electrolyte to form a new SEI film, thereby affecting ion transport and capacity loss. This impact was discussed in detail in subsequent experimental investigations.

The emergence of the relaxation voltage plateau typically depended on the precision of the detection equipment. Relaxation curves under different electrode SOC cutoff conditions revealed that the voltage plateau at high SOC occurred at earlier discharge rates (Figure S5). However, the earliest relaxation voltage plateaus across different discharge conditions all exhibited approximately 1.2% of the maximum plated lithium capacity (Figure S5d), indicating the lithium plating detection threshold of the differential relaxation voltage curve. Duan et al. [41] conducted similar experiments and simulations in full cells, reporting a safe charging boundary of 0.034 Ah. Their safety threshold (approximately 1.06% of battery capacity) aligned closely with the results obtained in this work. However, due to the smaller working area and differences in material properties, a relatively higher detection threshold was observed in half‐cells [45].

More importantly, the 1+1D model has recently been widely employed to investigate the relationship between macroscale kinetic parameters and capacity loss in batteries. However, due to its homogeneous structural approach, it could not predict the impact of heterogeneous structures (pores, active intercalation surface area, particle size, etc.) in actual cells on lithium plating‐stripping behavior. To elucidate this critical aspect, we extracted graphite particles with various morphologies from SEM images of the pristine electrode surface (Figure 3h). After scaling to the same area, these particles were applied to solve the lithium plating‐stripping kinetics equation in an extra dimension (model setup detailed in Methods 4.5). At 1C and 80% SOC, the circular graphite particle electrode (Circle) completed reversible lithium stripping in the shortest time (Figure 3i), while the electrode with the coarsest boundary particles (A3) required the longest stripping time. Particles with lower sphericity also exhibited longer stripping times, likely due to the detrimental effect of longer SSD distances on the insertion reaction. Yang et al. [38] found that the stripping rate during the relaxation period strongly depends on the insertion kinetics of the graphite electrode. As a sub‐reaction of the stripping reaction (Li(s) → Li^+^ + e^−^), the insertion reaction (Li^+^ + e^−^ + C_6_ → LiC_6_) was constrained at particles with longer SSD distances, leading to delayed stripping times. Consequently, the particle morphology assumed in the 1+1D model overestimated the kinetic rate of lithium plating‐stripping.

Additionally, we used SEM images of the electrode surface to roughly estimate the PSD (Figure 3j), thus revealing the influence of particle size. The PSD of the electrode surface exhibited a unimodal distribution (Figure S6a) with an average radius of approximately 8.5 µm and a PSD distribution ratio of about 2.5 (D_90_/D_20_). After discharging to 80% SOC, larger particle sizes exhibited less capacity loss (Figure 3k). Furthermore, particle morphology influenced the trend of dead lithium content with particle size, and A3 particles electrode with the roughest surface showed the most drastic variation with particle size. A potential reason was that larger particles possessed a smaller specific surface area, thereby weakening the lithium plating kinetic rate. Irregularly shaped particles exhibited more restricted insertion reaction kinetics, which were consequently more sensitive to variations in particle size. In summary, the 1+1D model might fail to provide accurate information regarding the influence of electrode structural morphology on lithium plating‐stripping behavior, which was considered a crucial determinant of battery electrochemical performance [24, 46].

### 2.3 Microstructural Resolved Modeling to Reveal Lithium Plating‐Stripping Behavior within Electrodes

#### 2.3.1 XCT for Characterizing the Microstructural Features of Graphite Electrodes

**FIGURE 4:**
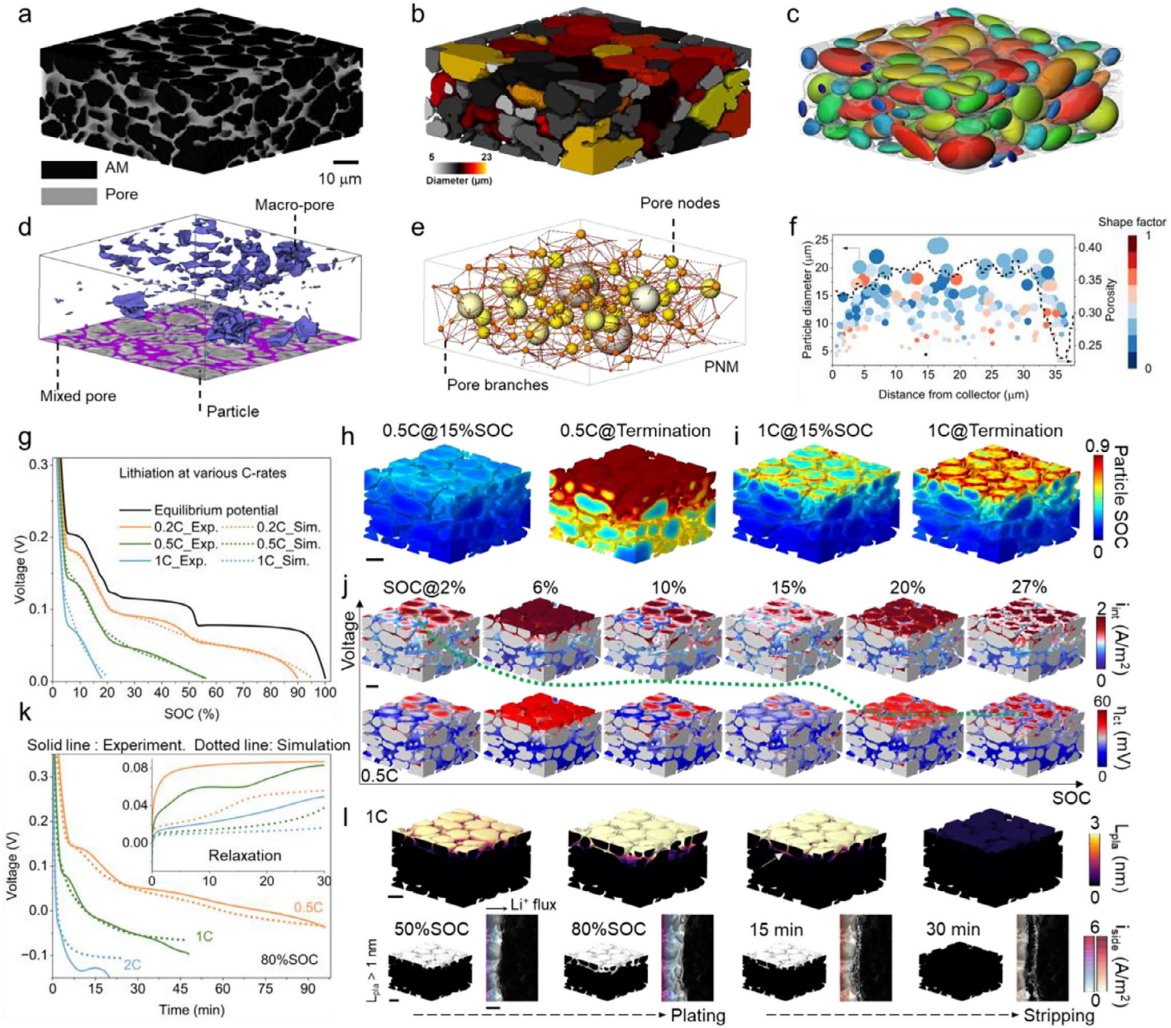
Microstructural resolved models reveal heterogeneous lithium deposition‐stripping behavior. (a‐f) Microstructural characterization of graphite electrodes. (a) 3D distribution of graphite solid phase/pore phase. (b) 3D spatial distribution of graphite particles. (c) Sphericity distribution of particles, with color dependent on particle size. (d) Distribution of macropores in the electrode. (e) Node‐network model of electrode pores, with color correlating to pore size and throat dimensions. (f) Distribution of particle size and in‐plane porosity across thickness. (g) Model‐predicted battery voltage results. (h,i) SOC distribution at the electrode particle level: (h) 0.5C, (i) 1C. (j) Relationship between electrode reaction rate and open‐circuit potential. (k) Model‐predicted lithium plating‐stripping voltage curves. (l) Dynamic evolution of lithium plating‐stripping during electrode operation. All scales in the figure represent an actual length of 10 µm.

To understand the heterogeneous morphology within actual graphite electrodes, the 3D microstructure of graphite electrodes was obtained using micro‐CT (Zeiss Xradia 610 Versa) with a spatial resolution of 0.25 µm (Figure 4a), and deep learning (DL) approach was employed to identify the particulate phases within the graphite electrodes (Figure 4b; detailed segmentation description was provided in Methods 4.4). Notably, conductive binder domains (CBD) within the electrode could not be distinguished in CT images due to their low X‐ray attenuation and nanoscale dimensions. Nevertheless, their low volume fraction (approximately 0.025) exerts a negligible influence on the electrode's active surface area and ion transport. This is demonstrated in the Note S1 through SEM images and virtual structures. Consequently, the presence of this phase is disregarded here. Highly heterogeneous particle sizes were randomly distributed throughout the electrode's 3D space. PSD results indicated that the D_50_ of the graphite electrode was approximately 11 µm (Figure S6b), showing significant discrepancy with the D_50_ (∼16.5 µm) identified from the electrode surface SEM image (Figure S6a). This discrepancy aroused because surface SEM could not resolve particle sizes deep within the electrode, and the surface of the electrode after rolling exhibited a near‐circular contour, which could be mistaken for the diameter plane of the particles. Also note that after the rolling process, most natural graphite spherical particles did not exhibit a spherical morphology (Figure 4c). The numerous flattened ellipsoidal particles represented a stark contrast to the assumption of spherical material particles in the 1+1D model.

Furthermore, most particles aligned their major axes parallel to the electrode plane, yielding a more intricate pore network structure. For instance, Figure 4d shows that the electrode contained only a limited number of macro‐pores, which were mostly oriented in parallel. Similar distribution patterns were also observed in additional samples imaged at 0.2 µm spatial resolution (Figure S7). These findings underscored how particle morphology and spatial organization influenced pore distribution within the electrode. In fact, the electrode possessed a higher proportion of micro‐pores, as indicated by the smaller pore nodes in the pore network model (PNM) (Figure 4e). The connections between these varied pore nodes created complex pore throats, which were likely to restrict mass transport and thereby impacted the cell's electrochemical performance [24, 34]. The abundance of macro‐pores observed on the electrode surface did not represent the electrode's overall volume fraction, as illustrated by the sharp variation in planar porosity near the separator in Figure 4f. An underlying cause was the migration of binders during electrode slurry evaporation, and the rise of microbubbles [47], and this migration rate strongly depended on drying temperature and rate. Fortunately, the random distribution of particle sizes as well as the sphericity confirmed the uniform dispersion of the graphite slurry, which facilitated uniform electrochemical kinetics on the electrode.

#### 2.3.2 3D Microstructure Model Reveals Lithium Electroplating‐Stripping Kinetics

A model for perceiving lithium plating‐stripping reactions was established based on the 3D graphite microstructure, with all model parameters obtained from the 1+1D model inputs (model parameters were detailed in the Supplementary Methods). Crucially, the 3D microstructure model considered the structural anisotropy of each component, enabling it to predict discharge voltage curves consistent with experimental results at various discharge rates (Figure 4g). Based on the discharge curves, both the capacity and voltage plateaus of the battery rapidly decreased with C‐rate increasing. At the microscopic level, during the initial discharge stage (15% SOC), graphite particles exhibited higher SOC as the rate increases from 0.5C to 1C (Figure 4h,i). However, due to larger particle size and ionic transport resistance [48], electrode overpotential increased rapidly at high current densities (Figure 4g), hindering the development of insertion reactions across the electrode thickness. At the end of 1C discharge, substantial unused material remained at the collector side, and large concentration gradients existed within particles near the separator (Figure 4i). In contrast, the electrode at 0.5C exhibited a relatively higher and more uniform SOC distribution (Figure 4h).

Interestingly, the insertion reaction at the electrode during operation was not a monotonically continuous process, but rather exhibited a pulsed behavior. This phenomenon was primarily reflected in the abrupt increase and decrease of the insertion current density i_int_ as the equilibrium potential underwent a sudden drop and subsequent stabilization (Figure 4j; Figure S9). For example, at 1C, when SOC increased from 2% to 6%, the equilibrium potential abruptly dropped, causing a sharp increase in current density. Subsequently, the equilibrium potential entered a plateau phase (SOC: 6%–15%) where the intense insertion reaction within the electrode gradually subsided. Later, with another abrupt drop in equilibrium potential, i_int_ increased again. Note that while the plateau in equilibrium potential represented coexistence stages of different phases in actual graphite electrodes (Stage 3L/Stage 2, Stage 2/Stage 1) [3, 49], the solid solution model primarily focused on its electrochemical implications for internal mass transport and reaction processes. This phenomenon was thus also termed the “insertion reaction wave” or “self‐equilibration” [50], a more detailed discussion is included in Note S2.

Lithium plating‐stripping voltage curves at different rates demonstrated the microstructure model's excellent predictive accuracy, though the model exhibits overestimated stripping plateaus and reduced open‐circuit voltages (Figure 4k; Figure S8). By incorporating particle size heterogeneity, the 3D microstructure model exhibits a heterogeneous particle size distribution and a smaller average particle size (Figure S6). This implies greater lithium plating reaction intensity and increased lithium insertion capacity at the same SOC, leading to an earlier onset of the stripping plateau and a lower voltage plateau. Despite these differences, the mechanism revealed by the model remains applicable to systems with similar parameters. A more detailed discussion of the comparison of simulation results from different models is provided in Note S3. Observing Figure 4k reveals that as the C‐rate increased, the voltage dropped prematurely below 0 V, and the stripping plateau during the relaxation phase became longer (Figure 4k). The internal state during 1C discharge was visualized using the model (Figure 4l). At 50% SOC, the plated lithium on the electrode surface had already exceeded 1 nm. By the end of discharge (80% SOC), the plated lithium had extended to the surface of the second layer of particles. However, no signs of plated lithium (white phase diagram) were ever observed deep within the electrode, likely due to LSD limitations, as indicated by the perspective view of the side reaction current density. The simulated lithium plating distribution and deposition current distribution were supported by similar phenomena captured by Duan et al. [41]. Results at other rates were comparable to 1C, but exhibited differences in the lithium plating onset SOC and the thickness of the deposited lithium layer (Figure S9).

During the stripping process, the reaction primarily occurred at the electrode surface. After the current interruption (15 min), only a slight reduction in the internal plated lithium was observed (white markings). As time passed, by the end of relaxation (30 min), the thickness of the plated lithium on the electrode had decreased to below 1 nm. However, the persistent stripping current density indicated that the deposited lithium was not completely stripped away, and a phenomenon was also observed at lower discharge rates (Figure S10). This reversible lithium would convert into dead lithium during subsequent cycles, leading to a decrease in coulombic efficiency (Figure 1d). Furthermore, at the particle scale, we observed that smaller particles tended to have less deposited lithium than larger particles (Figure S11). Distinguishing from the predictions of the 1+1D model, the assumption of a homogeneous PSD distribution implied the absence of reaction‐limiting processes within the electrode. In reality, electrodes with different PSDs exhibited reaction migration due to variations in specific surface area. This could be explained by smaller particles possessing larger specific surface areas, which reduced the current density during lithium plating [51, 52].

### 2.4 The Effect of Plated Lithium on the Physical and Electrochemical Properties of Electrodes

**FIGURE 5:**
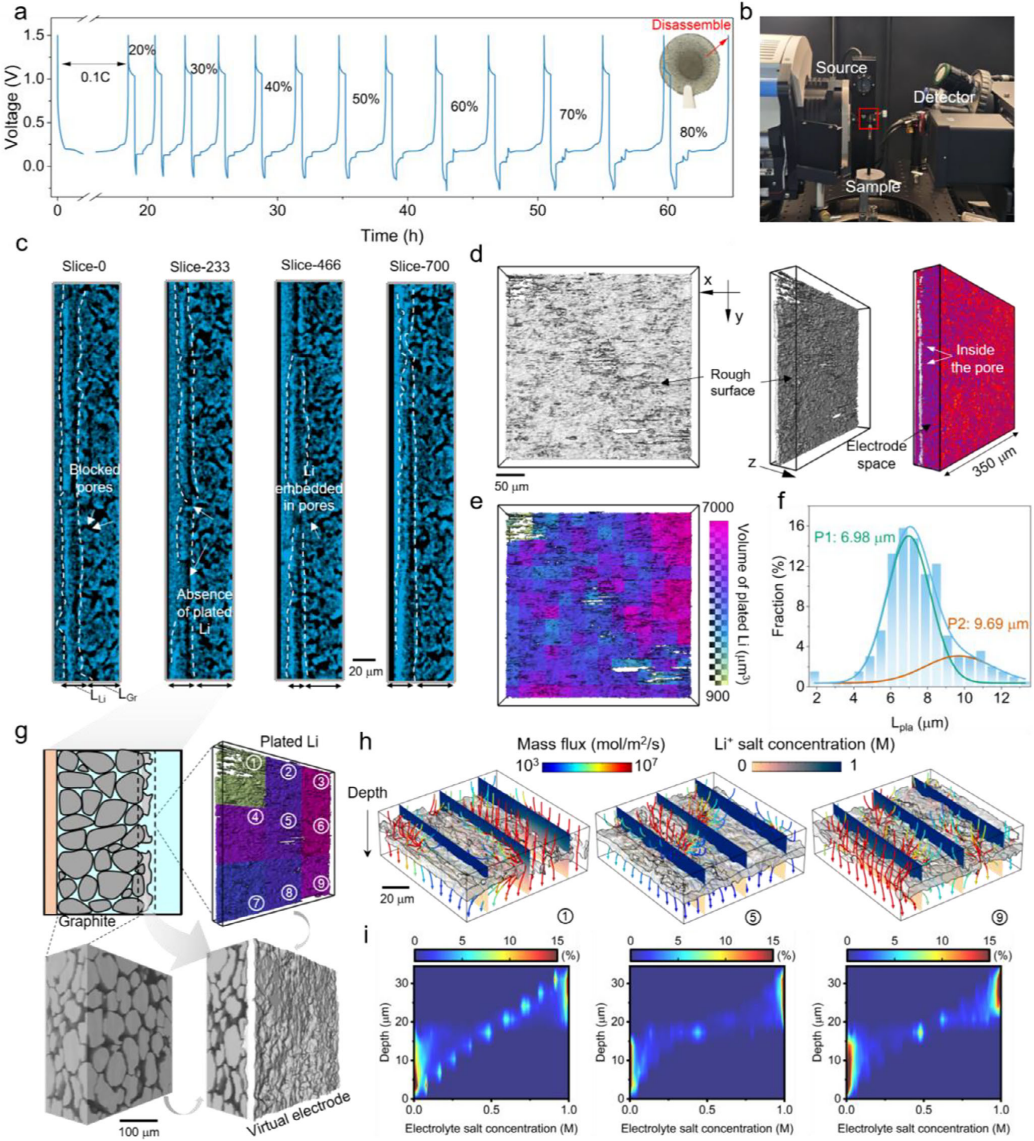
Effect of plated lithium on electrode performance. (a) Cycling conditions for plated lithium electrodes. (b) Ex situ imaging of disassembled electrode sheets using micrometer XCT. (c) Gray‐scale image slices of electrodes at different locations. (d) 3D image of internal plated lithium. (e) Thickness distribution across different regions of the plated lithium. (f) Quantification of coated lithium thickness across different regions. (g) Schematic illustrating numerical method for matching graphite electrode and plated lithium. (h) Effect of plated lithium in different regions on electrode LSD. (i) Quantification of salt concentration distribution at different thickness locations.

The continuous accumulation of plated lithium on electrode surfaces during cycling adversely affects both power performance and cycle life of batteries [2, 28]. However, this impact was typically diagnosed macroscopically using tools such as EIS or cyclic voltammetry (CV) based on data discrepancies [15, 16], leaving the microscopic mechanisms underlying these performance degradations unclear. For that purpose, the cell depicted in Figure 1c (with its cycling curve shown in Figure 5a) was disassembled, and the electrode piece was scanned using ex situ XCT with 0.5 µm resolution (Figure 5b), detailed XCT experimental procedures are provided in Note S4. Consistent with model predictions, the plated lithium was predominantly distributed on the electrode surface (Figure 5c). Since it was impossible to distinguish the contour of plated lithium within the graphite electrode, we analyzed only the plated lithium on the electrode surface here, despite its role as the primary barrier limiting LSD diffusion. Close observation revealed that the morphology of the plated lithium exhibited strong spatial heterogeneity. For instance, slice 233 and slice 466 showed discontinuities in the plated layer at localized positions (Figure 5c i,ii). Furthermore, the coarse white outlines also broadly indicated strong fluctuations in plating thickness across spatial locations. Nevertheless, the precious macropores on the separator side were largely covered by deposited lithium (Figure 5c i,iv).

The spatially non‐uniform plated lithium was further magnified in the 3D view (Figure 5d). The rough surface illustrated the spatially non‐uniform nature of the lithium plating‐stripping reaction, reflected in local defects and protrusions, the latter representing portions trapped within the graphite electrode pores. The localized absence of plated lithium might be related to the structure and material properties of graphite particles. For instance, smaller particles possessed larger surface areas, which could alleviate the burden of lithium plating during cycling (Figure S11) [53]. Figure 5e presents a quantitative visualization of the volume distribution of plated lithium across different regions. Only the upper‐right portion exhibited aggregated lithium plating, while the majority of the area showed significant spatial fluctuations in lithium plating thickness. Statistical data indicated that the majority of plated lithium thickness was around 7 µm, with another portion measuring approximately 10 µm (Figure 5f). This value appeared reasonable for cells that had experienced over ten cycles at a 2C rate [54].

The thicker plated lithium layer creates a curved transport pathway, requiring Li^+^ from the separator to reach the electrode surface with overcoming greater resistance at high rates. Additionally, the plated lithium adsorbed on the surface of graphite particles would block Li^+^ intercalation sites, thereby affecting the electrochemical performance of the electrode. Therefore, a virtual matching model of the graphite electrode and plated lithium was established (Figure 5g), with the model configuration detailed in the Supplementary Methods. The spatial non‐uniformity of plated lithium was reflected in differences in mass transfer resistance within the separator. Comparing the results of the three plated lithium regions in Figure 5h, the largely non‐plated area in Region ① provided more ion transport pathways, as evidenced by the increased streamlines. Conversely, the other regions exhibited more extensive plated lithium coverage over the electrode surface. Although ions could still pass through, this structure demonstrated a partitioned distribution of salt concentration above and below the plating layer. The plated lithium in the other regions also exhibited greater concentration gradients, particularly in Region ②, where ion transport was nearly blocked (Figure S12). The distribution of electrolyte salt concentration along the separator depth indicated that salt concentration tended to deplete with increasing depth (Figure 5i). In region ①, more transport channels enabled the mass distribution to exhibit a linear variation with depth. In contrast, for other regions containing more plated lithium, the salt concentration dropped sharply between 10 µm and 20 µm depth. Similarly, this phenomenon was observable in region ②, where the absence of channels caused salt concentration to vanish at the middle of the separator (Figure S12). It should be further noted that transport channels focused ionic currents at localized positions. During high‐rate operation of the cell, these locations inevitably exhibited strong potential gradients, leading to significant ohmic heating [55].

The obstruction of the reaction area by bulk lithium exacerbated the heterogeneous interfacial reactions within the electrode (Figure S13f), causing the electrode to experience an uneven lithiation process (Figure S13d). Simultaneously, the limited contact interface between the electrode and separator leaded to an uneven distribution of in‐plane ion concentration (Figure S13e). This heterogeneous electrochemical kinetics increased overpotentials within the cell, driving premature lithium plating reactions within the electrode (Figure S13a). The substantially increased lithium plating mass rendered the plated lithium more prone to fracture at the substrate, as quantified by the loss of capacity on the plated electrode, which was twice that of the pristine electrode (Figure S13a). Notably, the coverage of plated lithium increased the interfacial kinetic resistance, concentrating the lithium plating‐stripping reaction at the available area (Figure S13b,c). Through XCT imaging of plated Li in half‐cells and simulation of microstructural models, the uneven distribution of lithium plating reactions on graphite electrodes was revealed, which also appeared to explain the non‐uniform distribution of plated lithium on the anode surface after full‐cell disassembly [5]. Particle‐level electrode structure induced inconsistencies in Li plating reactions, while residual dead lithium consumed the electrode's active surface area, thereby exacerbating heterogeneous Li plating reactions. Furthermore, the larger surface area of graphite electrodes in full cells is more likely to cause in‐plane uneven electrode thickness, which is detrimental to uniform reactions. This inference process, supported by experimental and simulation results, is summarized in Note S5. Furthermore, we had not resolved the electrochemical processes governing the accumulation of lithium deposits on the plated layer, although this involved redistribution of ions and liquid‐phase concentrations.

### 2.5 Particle Size Regulation Mitigates Lithium Plating Side Reactions

#### 2.5.1 Microstructure Models Revealing the Influence Mechanism of Particle Size Regulation

Microstructural modeling and ex situ XCT revealed non‐uniform lithium plating‐stripping reactions on the electrode surface. Reflectively, particles of different sizes apparently exhibited variations in side‐reaction tendencies and intensity due to their physical characteristics, as reflected in the plated lithium thickness (Figure S11) and the rough profile of the plated lithium (Figure 5c–e). Therefore, a strategy for a graded electrode was inspired by arranging particle positions, i.e., utilizing the physical characteristics of different particles to improve the electrochemical side reaction kinetics of lithium plating [33, 48]. Here, a microstructure model was employed to investigate the effectiveness of this structural strategy and provide theoretical guidance for practical electrodes.

**FIGURE 6:**
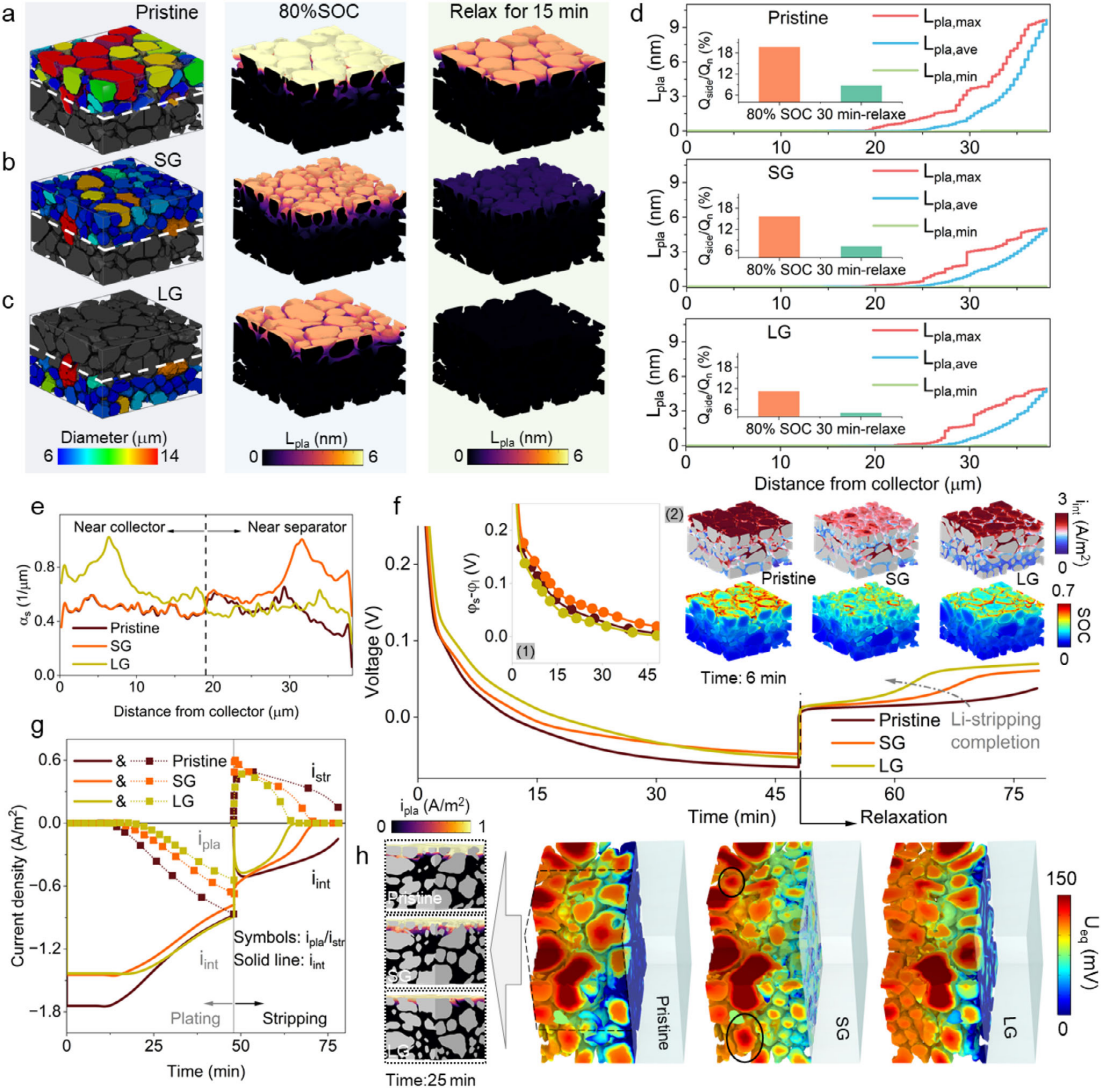
Microstructural models reveal the influence of particle size regulation on lithium plating‐stripping reactions at electrodes. (a–c) Evolution of plated lithium thickness during electrode operation: (a) pristine electrode, (b) SG electrode, (c) LG electrode. (d) Distribution of plated lithium along the electrode thickness at 80% SOC. The inset shows the lithium content at different time points. (e) Distribution of specific surface area along the electrode thickness. (f) Li plating‐stripping voltage curves at 1C rate. The inset shows (1) the average interfacial potential and (2) a 3D view of the operating state during operation. (g) Variation of current density during electrode operation. (h) Distribution of Li plating current and equilibrium potential within the electrode at the 25‐min time point.

Compared to the pristine electrode (Figure 6a), smaller particles on the separator/current collector side of the graded electrode were obtained via morphological erosion and random packing methods [56]. First, a graded electrode with small particles on the separator side (SG, Figure 6b) was prepared. Conversely, an electrode with small particles on the bottom layer was obtained by mirror‐flipping the SG electrode (LG, Figure 6c). The particle size distribution across the electrode thickness revealed that, to achieve the same AM fraction, a higher proportion of smaller particles were distributed on the separator side of the SG electrode or the current collector side of the LG electrode (Figure S14a). More importantly, compared to the pristine electrode, these smaller particles enabled the electrodes to achieve a larger specific surface area in the modified regions (Figure 6e).

The increased Li^+^ intercalation sites enhanced the electrode's power performance, as demonstrated by the discharge voltage curve at 1C (Figure 6f). Compared to the pristine electrode, the graded electrode exhibited varying degrees of overpotential reduction during discharge due to enhanced specific surface area and SSD. During relaxation, the graded electrode demonstrated a shorter lithium stripping plateau. Surprisingly, the LG electrode completed stripping earlier than the SG electrode, which was validated through visual observation of the thickness of the lithium deposition layer within the electrodes (Figure 6a–c). When discharging to 80% SOC, the graded electrode exhibited thinner plating thickness compared to the pristine electrode. Despite differences in particle size on the separator side, the plated lithium thickness on the first layer of particles was similar for both LG and SG electrodes. However, the SG electrode appeared to deposit more metallic lithium on the second layer of particles (Figure 6b). Figure 6d quantifies the plating thickness on the electrodes at 80% SOC. The lithium plating reaction on the SG electrode had extended to a depth of approximately 18 µm, while the LG electrode remained safe within the 22 µm depth range. Although the graded electrodes exhibited reduced lithium plating thickness compared to the pristine electrodes, the particle size control strategy did not improve the uniformity of lithium plating within the reaction zone. After 15 min of relaxation, the LG electrode had nearly completed stripping due to its thinner lithium deposition layer, while the SG electrode retained considerable residue on its surface (Figure 6b,c). In fact, after relaxation, the LG electrode still lost approximately 5% of its capacity. Fortunately, this represented a saving of about 2% compared to the SG electrode and about 3.6% compared to the pristine electrode (Figure 6d–f). These results demonstrated the LG electrode's superior capability in improving the lithium plating reaction.

Careful examination of the electrode discharge voltage curves revealed that the SG electrode exhibited a more pronounced initial voltage drop compared to the LG electrode (Figure 6f). Wood et al. [33] also reported similar rate performance and cycling performance results in their experiments, though they did not provide detailed theoretical support. Overpotential analysis revealed that the liquid‐phase ion overpotential in the SG electrode had been effectively mitigated (Figure S14b). This improvement originated from the uniform pore distribution achieved by the fine particles in the upper layer, as demonstrated by the ion flux distribution (Figure S14c). However, the SG electrode exhibited a higher SSD internal resistance than the LG electrode (Figure S14b). The smaller particles in the SG electrode possessed a larger specific surface area, enabling faster reaction kinetics that accelerated lithium saturation. This facilitated the penetration of the reaction front into the deeper, larger‐particle layer of the electrode (Figure 6f inset (2)). Also, this implied that all locations within the SG exhibit higher SOC. As the lithiation reaction progressed, particles deep within the electrode reached a state of lithiation saturation, leading to an increase in SSD internal resistance. Conversely, the disrupted ion flux on the LG electrode confined reactivity to the large‐particle surface region. The limited surface area failed to drive the reaction deep into the electrode, leaving substantial unused material that weakened the solid‐phase overpotential of the electrode particles (Figure 6f inset (2)). The above analysis was further supported by the interfacial potential (φ_s_—φ_l_) (Figure 6f, inset (1)). The interfacial potential of the electrodes during operation exhibited the relationship SG > Pristine > LG. According to the definition of interfacial overpotential, η_ct_ + U_film_ + U_eq_ = φ_s_—φ_l_, this indicated that both the equilibrium potential and interfacial overpotential on the SG electrode were in a loaded state, which might intensify the lithium plating reaction.

The subsequent flattening of the voltage curve stemmed from the lithium plating reaction [8, 41]. Based on the current density during electrode operation, the SG electrode suffered lithium plating earlier than the LG electrode (Figure 6g). Due to the identical lithium plating rate, the SG electrode naturally developed a higher plated lithium thickness (Figure 6d). Although the regions reaching lithium plating reaction conditions (η_Li_ < 0 V) were concentrated near the separator (Figure 6h), the SG electrode exhibited a larger area for lithium plating reactions. We equally attributed this phenomenon to the fast kinetics of the SG electrode. The rapid lithiation kinetics of small particles on the surface layer of the SG electrode allowed the reaction front to propagate deep into the electrode in advance. Simultaneously, the current distribution in the intercalation reaction followed a “self‐balancing” principle, that is, the current density is shared between the small particles on the electrode surface and the larger particles within the electrode [13, 55]. At such high current densities, large particles with extended SSD distances lost their lithiation driving force rapidly, reflected in the larger equilibrium potential gradient within the particles (Figure 6h). Consequently, the larger overpotential drove the parallel branching of lithium plating currents within the electrode. Benefiting from the larger specific surface area of the reactive region (Equation 2), the lithium plating current rapidly accumulated plated lithium at the electrode surface.

The modification of pore structure and reaction kinetics by the SG electrode led to more severe performance degradation. Unlike the cathode side, where only the enhancement of battery power performance was considered [34], the negative impact of lithium plating reactions appeared particularly significant on the anode side. Conversely, LG electrodes exhibited decreased internal resistance due to limited reaction areas and smaller specific surface areas. Although LG electrodes avoided larger side reactions, the underutilization of materials remained a major challenge for enhancing their power capacity. Therefore, synergistic strategies to simultaneously improve electrode power performance and mitigate the hazards of lithium plating still required further investigation.

#### 2.5.2 The Role of Particle Size Control Strategies in High Energy Density Electrodes

**FIGURE 7:**
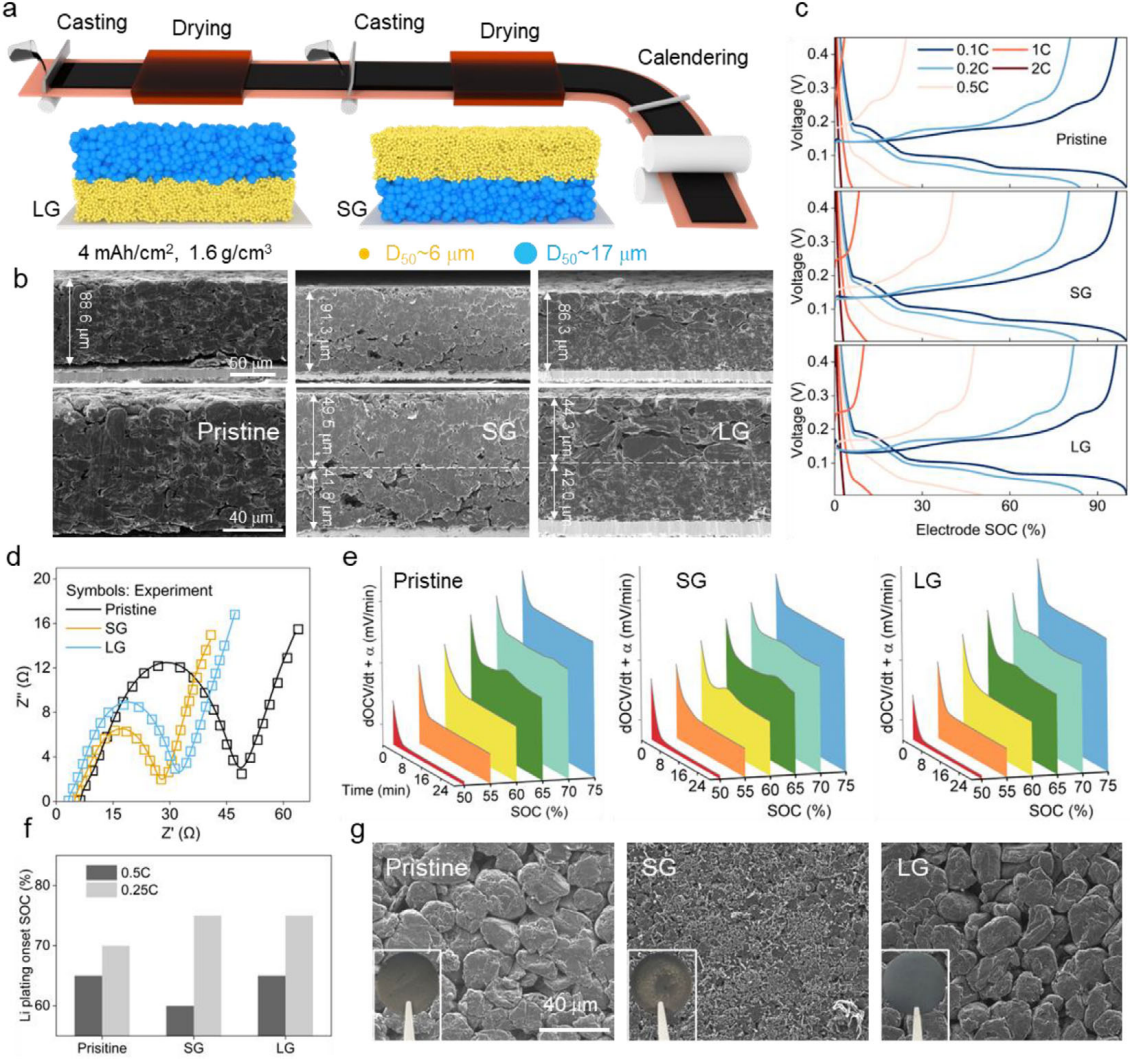
The effect of particle size regulation strategies in high‐energy‐density electrodes on lithium plating initiation conditions. (a) The experimental process for fabricating high‐energy‐density electrodes. (b) (Magnified) cross‐sectional SEM images of electrodes with different structures. (c) Rate performance of the electrodes. (d) Determination of lithium plating onset at 0.5C using differential voltage curves. (e) EIS impedance curves of the electrodes. (f) Onset SOC of lithium plating at different rates. (g) SEM images of the electrode disassembly after lithiation to 80% SOC at 0.5C.

Figure 7 employs a microstructure‐based model to investigate the impact of particle size regulation on lithium plating, revealing a competition phenomenon between the lithium plating reaction zone and the plating reaction rate. Due to liquid diffusion limitations affecting the electrodes, the lithium plating reaction occurs on the side of the separator. Based on this same performance constraint, we note that high‐energy‐density electrodes suffer severe liquid diffusion limitations due to their greater thickness and lower porosity, thereby hindering their commercialization. Although increasing the electrode thickness alters the structural parameters of the electrode, we demonstrate that under identical diffusion‐limited conditions, the results from the microstructure model will yield the same conclusions. This is discussed in the Note S6 and will be demonstrated in subsequent experiments.

4 mAh/cm^2^ dense electrodes were used to examine the impact of particle size distribution strategies on power performance and lithium plating side reactions. For this purpose, two natural graphite spherical powders with distinct particle size distributions (D_50_ approximately 6 and 17 µm, respectively) were selected. They were blended into a homogeneous slurry using a dry mixing process [57], and graded electrodes (LG and SG electrodes) were obtained through a two‐step casting‐drying method. Electrodes formed from large‐particle graphite powder served as reference objects (Pristine electrodes). These electrodes were rolled to achieve identical compaction density (∼1.6 g/cm^3^) and volumetric energy density (Figure 7a). Following secondary drying, the electrodes were transferred to a glove box and left to rest for one day to release roll‐compaction stresses. Qualified electrode sheets within a 0.5 mg and 3 µm tolerance range were selected for microstructural characterization (further error analysis is discussed in the Note S7). SEM cross‐sectional images revealed a distinct particle size distribution along the electrode thickness direction (Figure 7b). Notably, the SG electrode exhibited a crowded arrangement of small particles in the upper layer at higher packing densities, which fractured the pores into numerous micro‐pores. In contrast, the upper regions of the pristine and LG electrodes displayed a loose pore distribution. Beyond introducing manufacturing errors, the mutual repulsion between particles caused the electrodes to expand to varying degrees after rolling, particularly following the high‐temperature drying, leading to further variations in electrode thickness and porosity. The abundant pores between large particles in the upper layer of LG electrodes significantly mitigated thickness changes resulting from repulsion between smaller particles.

Distinct pore and particle distributions might remarkably influence electrode rate performance, as shown in Figure 7c. The introduction of fine particles in the graded electrode mitigated SSD polarization, resulting in superior performance compared to the pristine electrode at discharge rates exceeding 0.5C. However, the SG electrode exhibited lower discharge capacity than the pristine electrode at lower rates and demonstrated poorer cycling stability (Figure S15a). This differed from the results of simulations for thinner electrodes (Figure 6h). Therefore, EIS testing was conducted on three cells (Figure 7d), with fitted circuits and results presented in Table S1. The results indicated that the impedance of the gradient electrode was markedly lower than that of the pristine electrode. The fine particles at the bottom layer of the LG electrode increased the contact area between the electrode and the current collector, reducing the electrode contact resistance (R_0_) by approximately 63%. Due to the formation cycle and the use of a compatible electrolyte, the SEI film resistance on the electrodes is minimal. The SEI film resistance relative to the charge transfer resistance is low for each electrode, resulting in an initial SEI film resistance of approximately 10 nm as obtained from model fitting (Table S1). As demonstrated by the simulations in Note S8, the SEI film resistance remains extremely low, even negligible, under various structural conditions. Therefore, based on SEM images (Figure 7b), we attribute the enhanced performance of the SG electrode to the use of smaller particles than those in the microstructural model, which enables an immediate and significant increase in capacity. Similarly, this reduced solid‐state diffusion further facilitates the expansion of the reaction area, thereby promoting the occurrence of the lithium plating reaction. As current increases, all electrodes exhibited LSD‐dominated characteristics. Within the highly confined active reaction area, the large SSD resistance and charge transfer resistance of the pristine electrode led to rapid battery failure. In contrast, the gradient electrode gained capacity enhancement due to reduced interfacial resistance or SEI film resistance. Although the fast reaction kinetics of the SG electrode compensated for the thicker SEI film, the extended reaction area activated the SSD limitation of large particles at the bottom layer of the SG electrode (Figure 6f inset (2)), preventing the SG electrode's capacity from exceeding that of the LG electrode.

Similar effects were reflected in the onset SOC performance of lithium plating (Figure 7e). At 2 mA/cm^2^, contrary to simulation results, the SG electrode exhibited poor tolerance for lithium plating reactions (60% SOC). This was because the extended reactive area of the SG electrode increased the overall overpotential of the electrode, while the reaction kinetics of small particles were hindered by the SEI film [58], leading to an early onset of lithium deposition. Meanwhile, the larger specific surface area of the upper layer's smaller particles accelerated the lithium plating reaction. In contrast, the pristine electrode, with its smaller active area and lower specific surface area of larger particles, exhibited the same lithium plating onset SOC (65% SOC) as the LG electrode at 0.5C. Optical microscopy and SEM images of the electrode surface at 80% SOC revealed extensive lithium dendrites on the SG electrode surface, while no lithium plating side reactions were observed on the pristine or LG electrodes (Figure 7g). These results corroborated the analysis in Figure 6, i.e., the extended active area of the SG electrode led to an earlier onset of lithium plating, while the rapid surface reaction kinetics catalyzed a higher lithium plating rate. At lower current densities of 1 mA/cm^2^ (Figure 7f; Figure S15b–d), the reactive zone extended deeper into the electrode. At this point, the LG and SG electrodes demonstrated the advantage of graded electrodes in kinetic regulation (75% lithium plating onset SOC), while the pristine electrode exhibited an earlier lithium plating onset time (70% SOC) due to greater SSD polarization.

Therefore, for high‐energy‐density electrodes, operating conditions at different currents induced competition between kinetic rates and active reaction areas. Simulation of the SEI film and EIS testing demonstrated the negligible influence of the SEI film, enhancing consistency between microstructure model results and experimental validation. In summary, this work provides structural optimization recommendations through microstructure modeling to guide practical experimental applications. By ensuring model accuracy, elucidating lithium plating reaction mechanisms, and investigating reaction heterogeneity in 3D heterostructures, this study reveals that plating reactions exhibit both in‐plane and thickness‐dependent nonuniformity. This leads to proposed particle size tuning strategies, yielding competitive electrode architectures. The framework for this work is summarized in Figure S17.

## 3 Conclusion

Online monitoring of lithium plating onset and development faces challenges from equipment precision, material consistency, and physical understanding. This work established a multiscale electrochemical model based on electrode microstructure imaging to predict lithium plating‐stripping processes in experimental samples and elucidate the mechanisms of structural regulation.

The widely used differential voltage curve method has been shown to establish ambiguous lithium plating safety boundaries, allowing batteries to undergo hidden accelerated aging and mechanical short‐circuiting processes within erroneous SOC ranges. 1+1D model based on key electrode physical characteristics could identify critical lithium plating‐stripping events within voltage histories. The half‐cell model revealed the aggregation of lithium plating‐stripping reactions at the separator interface and quantified the 1.2% capacity detection threshold for dOCV/dt voltage profiling. The 3D microstructure‐resolved model reconstructed the heterogeneous material distribution within graphite electrodes, revealing heterogeneous kinetic processes across different scales. Importantly, differences in lithium plating‐stripping kinetics at the particle scale were observed. Smaller particles suppressed side reaction development due to their larger specific surface area. The inhomogeneous distribution of lithium metal plating was first revealed through ex situ XCT imaging. The model demonstrated that plated lithium blocks pores and covers active surfaces, leading to a deficiency in salt concentration supply within the electrode and accelerating the growth of plated lithium.

Additionally, the influence of graded electrodes on lithium plating‐stripping side reactions was numerically examined. LG electrodes with small particles arranged on the current collector side reduced SSD polarization while confining reactivity to a smaller area, thereby suppressing lithium plating reactions. The SG electrode with small particles arranged on the separator side diffused the reaction front deep into the electrode, creating a relatively early and larger lithium plating reaction zone. Experimental results further demonstrated that the small particles within SG electrodes exhibited rapid kinetic rates in thicker and denser electrodes, accelerating lithium plating during high‐rate conditions dominated by LSD. LG electrodes have been proven to ensure superior rate performance and resistance to lithium plating under various conditions. This combined multiscale modeling and experimental work provided a mechanistic understanding of lithium plating prediction methods. When integrated into battery management systems, the model would display the progression of lithium plating‐stripping processes in real time, supporting the optimized design of electrode structures and charge–discharge protocols.

## 4 Method

### 4.1 Materials

Graphite electrodes with a thickness of approximately 40 µm were acquired from Hefei Kejing for model development and validation, featuring an areal loading of about 2.2 mAh/cm^2^. To evaluate the efficacy of particle size control strategies in regulating lithium plating‐stripping reactions for high energy density electrodes, the loading of the graded electrode was increased to 4 mAh/cm^2^, and its porosity was rolled to achieve higher volumetric energy density. Natural spherical graphite particle power with a D_50_ of 17.4 µm was used to prepare a 4 mAh/cm^2^ single‐layer large‐particle electrode (Pristine electrode). Powders with D_50_ of 6 µm and 17.4 µm were used to prepare 4 mAh/cm^2^ particle‐graded electrodes. The active material (95 wt.%), conductive carbon (1 wt.%), CMC dispersant (1.5 wt.%), and SBR binder (2.5 wt.%) were uniformly mixed in a vacuum planetary homogenizer (ITT‐160S, INTEGRITY). The slurry was then coated onto 14 µm ‐thick copper foil. After drying for 1 h in a 60°C drying oven, the electrode sheet was rolled using a roller compactor (MSK‐2150, KEJING) to achieve a density of approximately 1.6 g/cm^3^ (porosity of about 26%). Subsequently, the electrode sheet was dried under vacuum overnight at 110°C. The graded electrode was prepared using a bilayer‐coating method. First, a single‐layer electrode sheet with an areal capacity of 2 mAh/cm^2^ was prepared. The second layer was coated onto the dried single‐layer electrode, followed by a secondary drying process and identical roll‐pressing steps. Figure 7a illustrates the manufacturing process.

### 4.2 Cell Assembly and Electrochemical Testing

The electrodes were cut into 12 mm diameter discs and dried again for 2 h, then they were transferred to argon‐filled glove box for assembling the Li||Gr half‐cells. The half‐cell consisted of a 16 mm Celgard 2325 separator, a 15.8 mm lithium metal foil, and 60 µL electrolyte (1.0 m LiPF_6_ in EC:EMC = 3:7 wt.% with 2.0% VC). After over 12 h of electrolyte wetting, the cells performed three formation cycles on a microcurrent tester (LANHE, M340A) to form a stable SEI film. Specifically, the cell was discharged at a constant current (CC) rate of 0.1C to 5 mV, then held in constant voltage (CV) mode until the current dropped to 1/40C. It was subsequently charged at 0.1C to 1.5 V to release the material's capacity. After formation, the cells were first subjected to a 0.1C CC‐CV‐CD cycle for capacity and nominal rate evaluation, followed by discharge at various rates to different SOCs using a constant step of 5% SOC. After reaching the target SOC, the cells were rested for 30 min to record the OCV vs. Li/Li^+^ voltage curve during relaxation. Subsequently, they were charged at 0.2C to 1.5 V to remove residual capacity (Figure S1).

The EIS test of the half‐cell was conducted on an electrochemical workstation (CHI660e). First, the graphite anode was lithiated to 50% capacity at 0.1C. Following a relaxation period exceeding 4 h, the cell was tested with a 5 mV voltage amplitude and a frequency range from 10 mHz to 1 MHz.

### 4.3 Postmortem Characteristics

After cycling at different SOC, the cells were transferred to a gearbox for disassembly. Graphite electrodes were repeatedly washed three times with dimethyl carbonate (DMC), and their surface and cross‐sectional morphologies were examined using SEM. For samples with lithium metal morphology, all specimens were sealed in an inert gas environment to avoid reaction with air before SEM and energy‐dispersive spectroscopy (EDS) characterization.

### 4.4 X‐Ray Computed Tomography and Image Analysis

Graphite electrode samples were cut to approximately 0.5 mm dimensions and mounted onto sample holders using transparent UV adhesive. The X‐ray micro‐CT system (Zeiss Xradia 610 Versa, Carl Zeiss Inc.) operated at a tube voltage of 90 kV (tungsten emission) and a power of 14 W. At 40× objective magnification, the distance between the X‐ray source and detector relative to the sample position was adjusted to achieve different voxel resolutions (0.2, 0.25, 0.5 µm). The corresponding field of view was approximately 1024 times the voxel resolution. Subsequently, approximately 2001 projections were collected over a 180° sample rotation angle. The exposure time per projection was set to approximately 2–7.1 s based on the spatial resolution. Projections were then corrected and reconstructed using the instrument's native software (XM Reconstructor, Carl Zeiss Inc.).

Graphite electrodes with plated lithium were taken out of the glove box and scanned with a spatial resolution of 0.5 µm. Note that the plated lithium was exposed to air for 2 h and 40 min during scanning. Although the morphology of the plating might have changed, as reported in some studies, these effects were very limited [59, 60].

The gray‐scale images obtained from scanning underwent positional correction and region‐of‐interest cropping in Avizo software (ThermoFisher Scientific), followed by segmentation of the particle phase using the U‐net deep learning (DL) module. First, three slices were manually selected along the electrode thickness direction to mark the AM region as masks. This approach was adopted because graphite exhibits low X‐ray absorption, while high‐density copper foil significantly affects X‐ray penetration, resulting in substantial contrast attenuation in the grayscale image along the thickness direction (Figure S16a). The masks were then used as training target input for the 3D U‐Net network. Under the defined two‐phase regions (AM phase + pore phase), the maximum training iterations were set to 200, with a learning rate of 1 × 10^−4^ and a batch size of 100. The trained model was used to automatically segment the image sequence. Figure S16b,c compares the results of grayscale threshold segmentation with DL segmentation. For the electrodes used in this work to develop the model, the active material has a weight fraction of 94.5 wt.% and a porosity of approximately 0.35. Calculated based on the material density (ρ_Gr_ is approximately 1.35 g/cm^3^ and ρ_CBD_ approximately 1.75 g/cm^3^), the volume fraction ratio of graphite to CBD is about 24.7, resulting in a CBD volume fraction of approximately 0.025 of total volume. This small volume fraction implies a negligible effect on electrode performance, with a more detailed argument provided in the Note S1.

Additionally, the pore network model (PNM) and moment‐of‐inertia‐dependent sphericity in Figure 4 were calculated using the label analysis module within the software. The segmented porous structure was converted into grayscale data, and adaptive finite element meshing was performed on the graphite microstructure using Simpleware software (Synopsys, Inc., Mountain View, USA). To demonstrate the volumetric representativeness of the selected regions and mesh independence, the structural parameter characterization and simulation results for different regions are discussed in the Note S9.

### 4.5 Microstructure‐Based Electrochemical Model

The microstructure of the graphite electrode obtained from XCT was segmented into an active particle phase and a pore phase (Figure 4). Compared to the 1+1D model, the 3D microstructure model identified the heterogeneous size distribution and morphological characteristics of pores and particles, thereby avoiding the estimation of physical parameters such as the overall porosity e∼, average particle size, and specific surface area (detailed model description is provided in Supplementary Methods). Additionally, the 3D reconstructed structure incorporated information from a larger volume range of the electrode, providing more accurate statistics on particle size compared to SEM surface images. Unlike the ensemble domain of the 1+1D model, the interface‐driven sources in the microstructure model were physically resolved geometrically [61]. Furthermore, in Section 2, particles with distinct morphologies based on SEM planar images served as fundamental units composing the electrode AM domain. Following steps such as particle contour identification, meshing, and dimensional scaling, the geometry was computed as a 2D region in extra dimensions to simulate solid‐state lithium diffusion processes.

## Funding

This work was supported by the LiaoNing Revitalization Talents Program (Grant No. XLYC2403079), the National Natural Science Foundation of China (Grant No. 52176058).

## Conflicts of Interest

The authors declare no conflicts of interest.

## Supporting information




**Supporting File**: advs74829‐sup‐0001‐SuppMat.docx.

## Data Availability

The data that support the findings of this study are available from the corresponding author upon reasonable request.
