# Supplementary material for: Microstructure‐Resolved Modeling to Predicting and Regulating Lithium Plating‐Stripping Dynamics on Graphite Electrodes
Source: Adv Sci (Weinh). 2026 Mar 25;13(30):e24109. doi: 10.1002/advs.202524109 (PMC13248799; doi:10.1002/advs.202524109)
Supplement: Supplementary file 1 — Supporting File: advs74829‐sup‐0001‐SuppMat.docx. [file ADVS-13-e24109-s001.docx]

**Microstructure-Resolved Modeling to Predicting and Regulating Lithium Plating-Stripping Dynamics on Graphite Electrodes**

Heng Huang^1^, Yang Li^1^, Xinyu Liu^1^, Zhifu Zhou^2^, Wei-Tao Wu^3^, Lei Wei^4^,

Chengzhi Hu^1^, Linsong Gao^5^, Yubai Li^1*^, Yongchen Song^1*^

1. Key Laboratory of Ocean Energy Utilization and Energy Conservation of Ministry of Education, Dalian University of Technology, Dalian 116024, China

2. State Key Laboratory of Multiphase Flow in Power Engineering, Xi'an Jiaotong University, Xi'an 710049, China

3. School of Mechanical Engineering, Nanjing University of Science and Technology, Nanjing 210094, China

4. Department of Mechanical and Energy Engineering, Southern University of Science and Technology, Shenzhen 518055, China

5. School of Mechanical Engineering and Mechanics, Xiangtan University, Xiangtan 411105, China

*Corresponding author: liyubai2021@126.com, songyc@dlut.edu.cn

**Supplementary Figures**


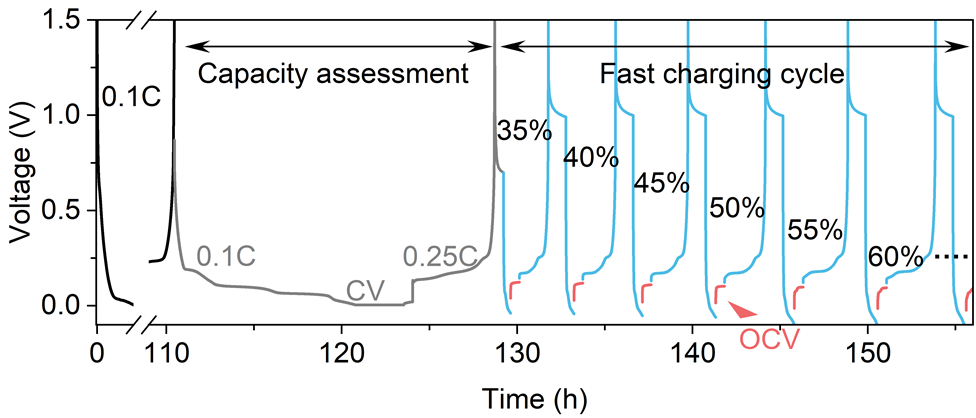


Figure S1. Electrochemical experimental procedure for monitoring lithium plating in an online Li||Gr half-cell.


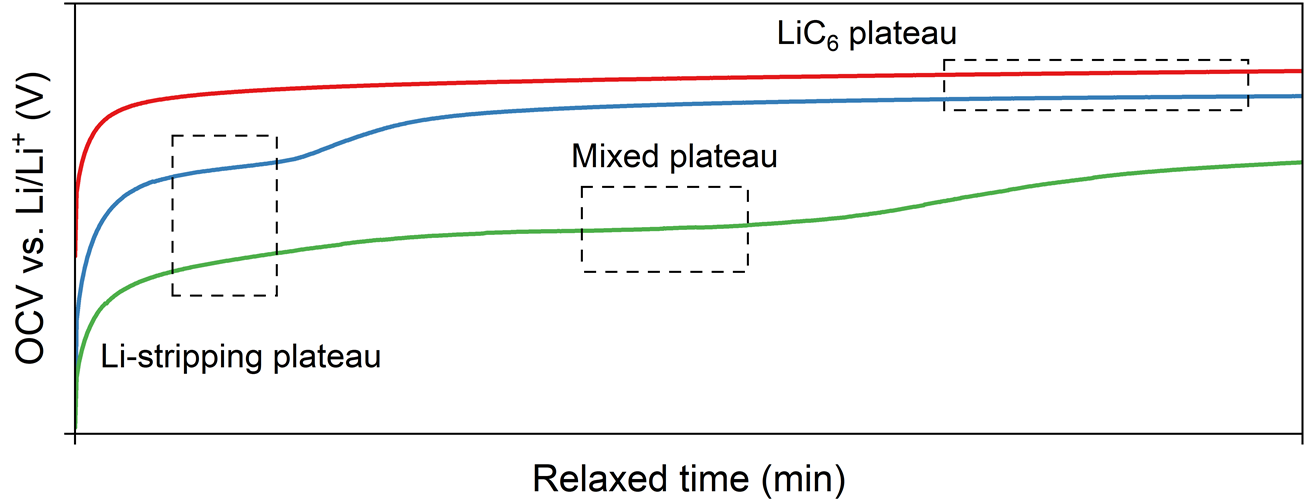


Figure S2. A method for detecting lithium plating by relaxation voltage after battery discharge.


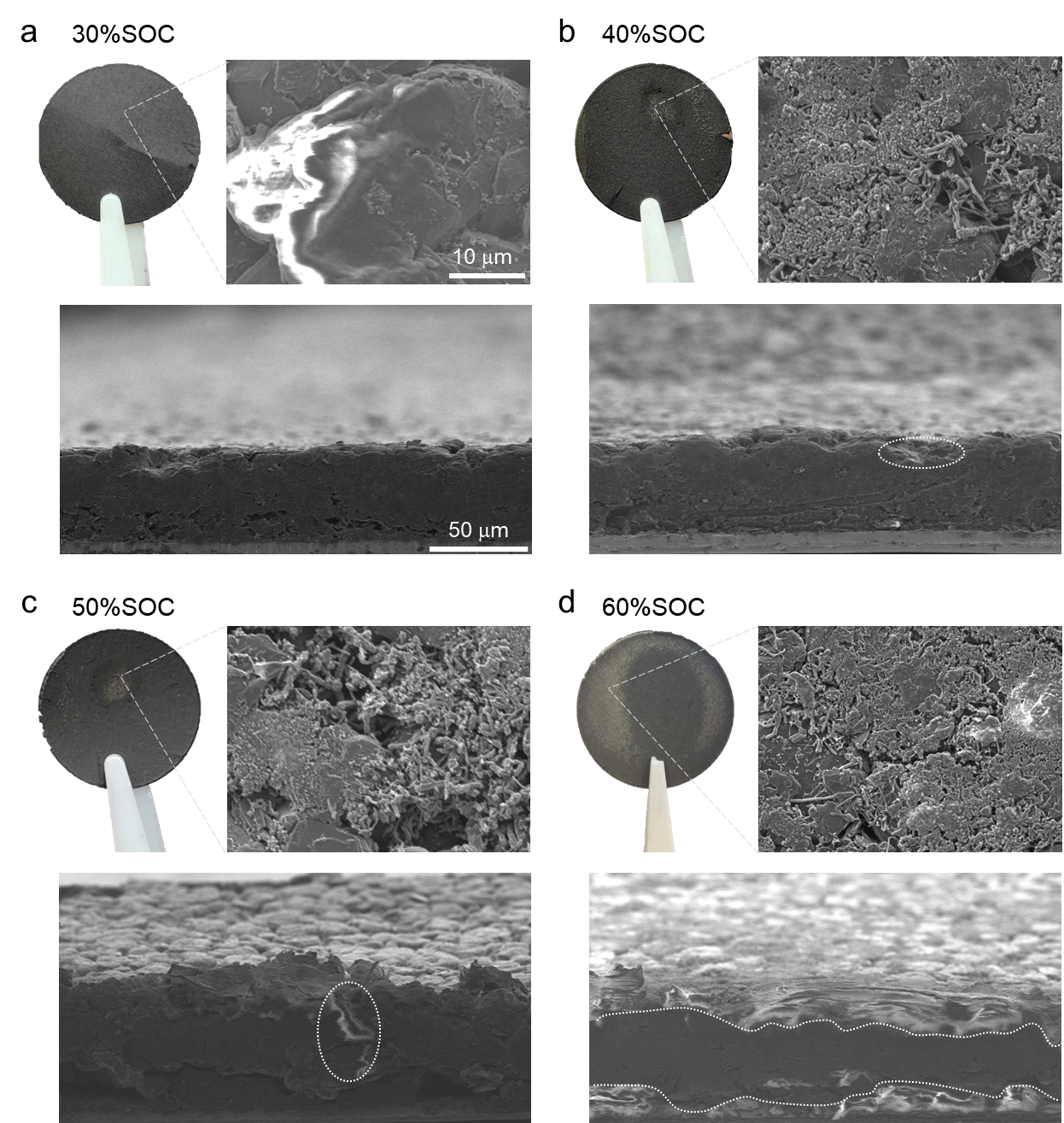


Figure S3. Surface and cross-sectional images of the graphite electrodes after cycling were used to examine lithium plating. (a) 30% SOC, (b) 40% SOC, (c) 50% SOC, (d) 60% SOC.


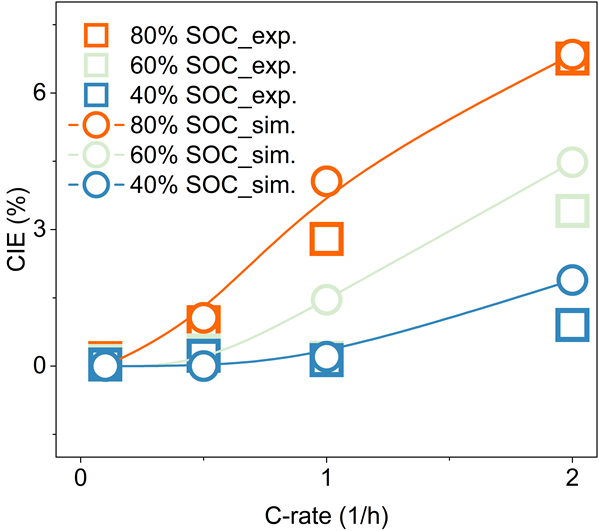


Figure S4. Comparison of CIE results tested in Li||Gr half-cells with predictions from the 1+1D model.


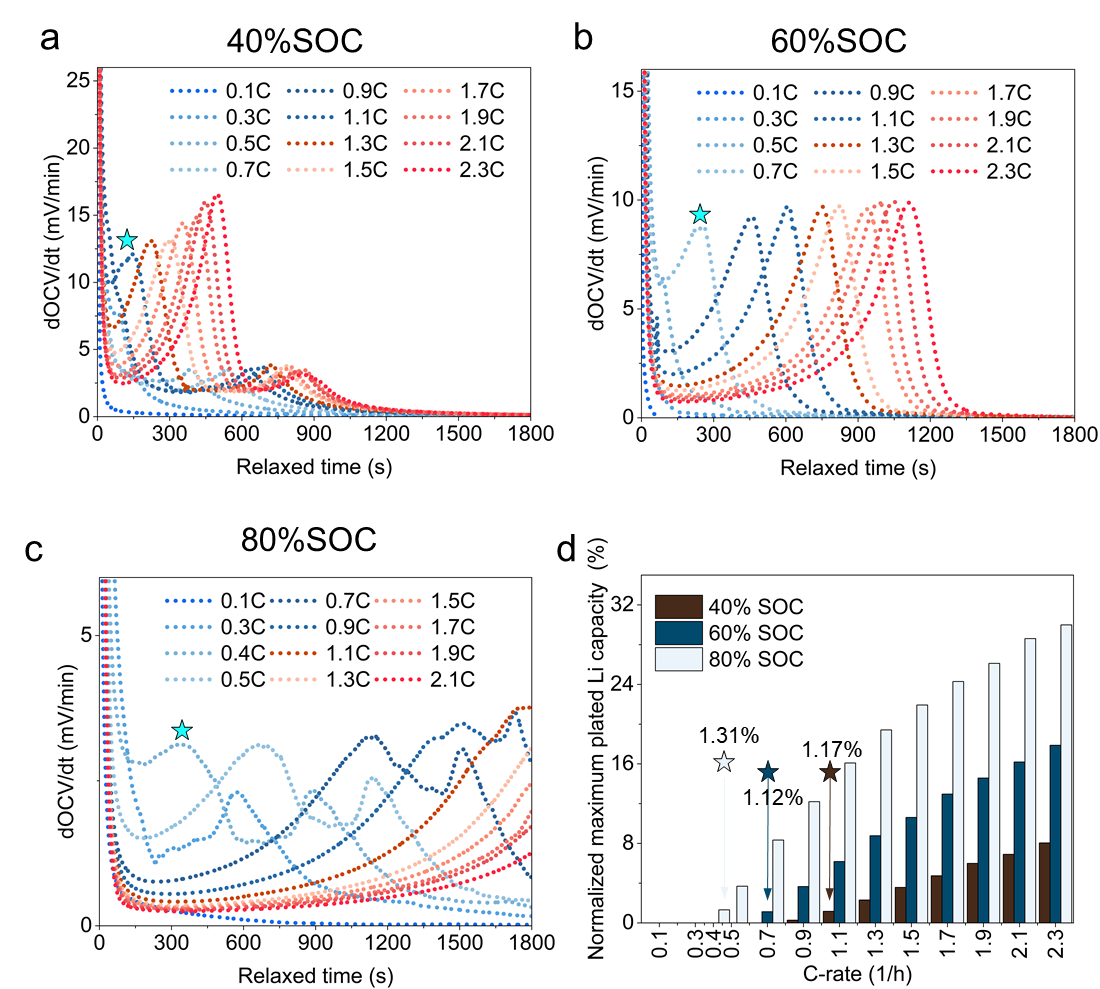


Figure S5. 1+1D model for identifying relaxation voltage plateau detection thresholds at different SOCs and discharge rates. Relaxation voltage differential curves under various discharge rates at (a) 40% SOC, (b) 60% SOC, and (c) 80% SOC. (d) Histogram for identifying voltage plateau detection thresholds.


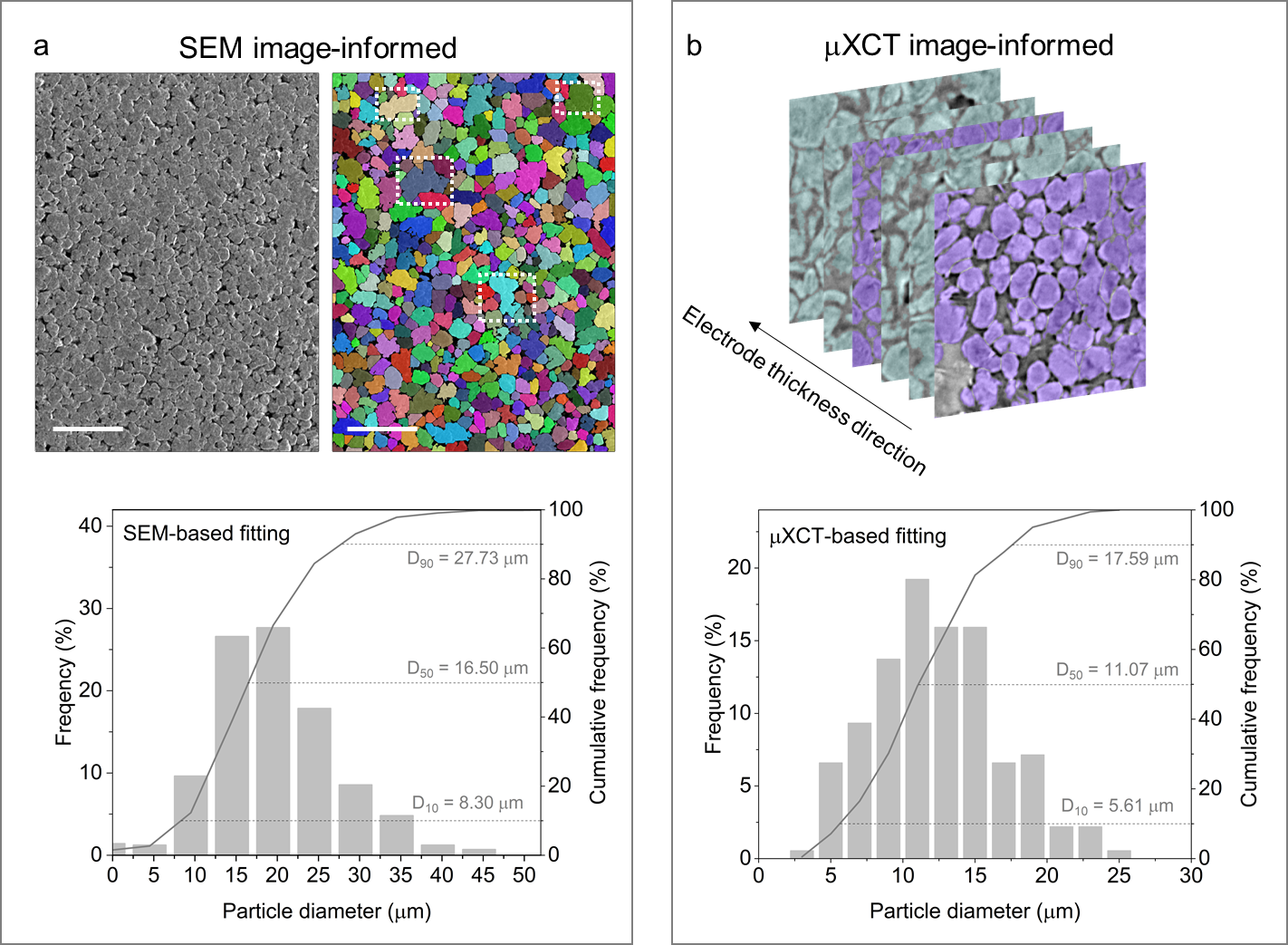


Figure S6. PSD of graphite electrodes based on image recognition. (a) PSD of surface particles on graphite electrodes derived from SEM images. (b) PSD of graphite electrode particles derived from XCT images.


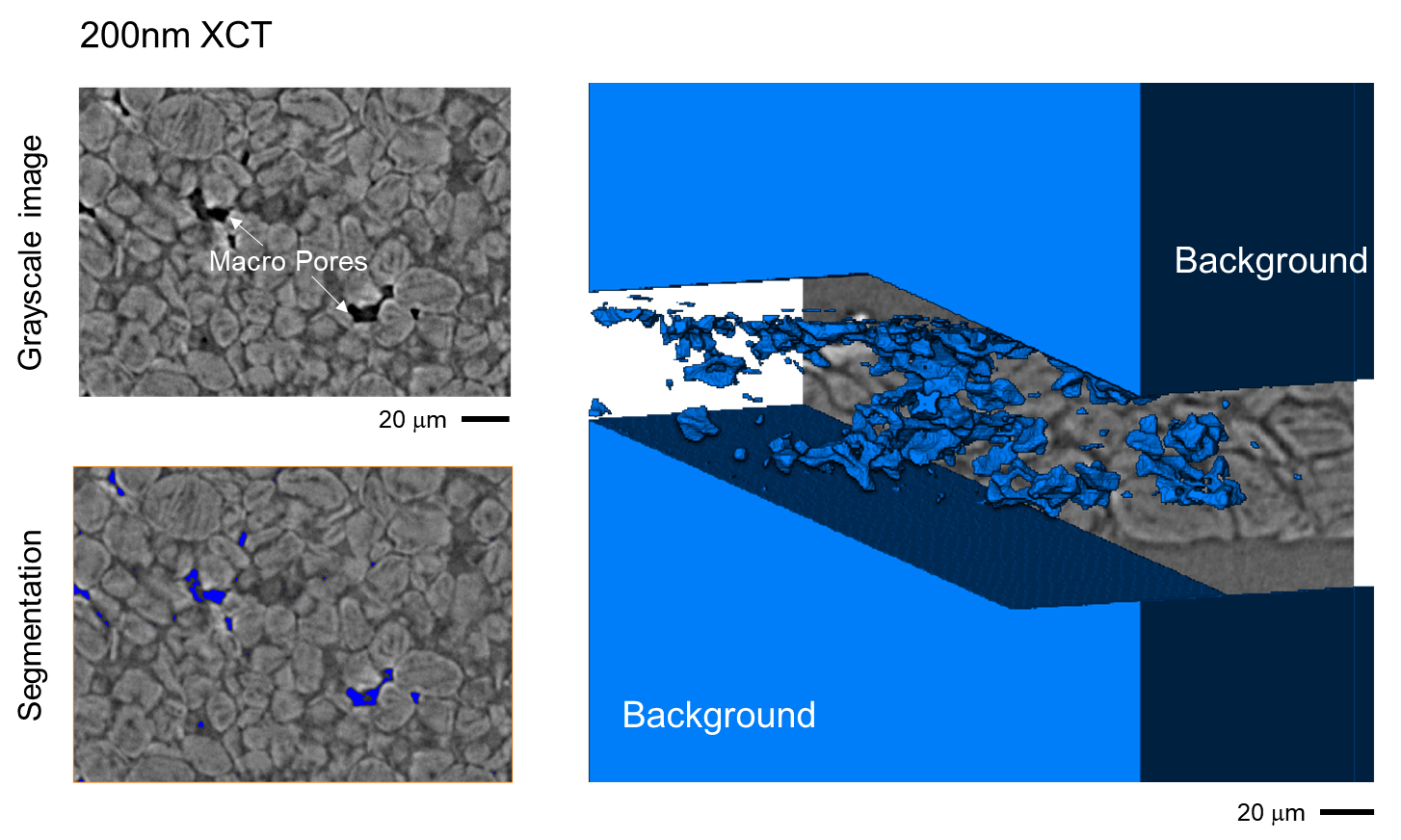


Figure S7. Micron-scale CT scans of graphite electrodes with 0.2 μm spatial resolution were employed to distinguish macro-pores.


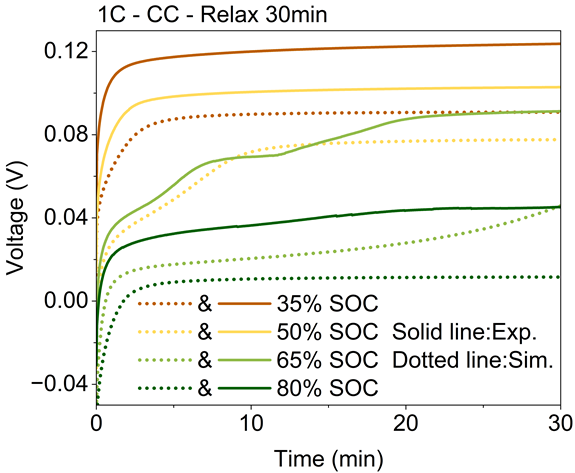


Figure S8. Relaxation voltage curve of the cells predicted by the microstructure model.


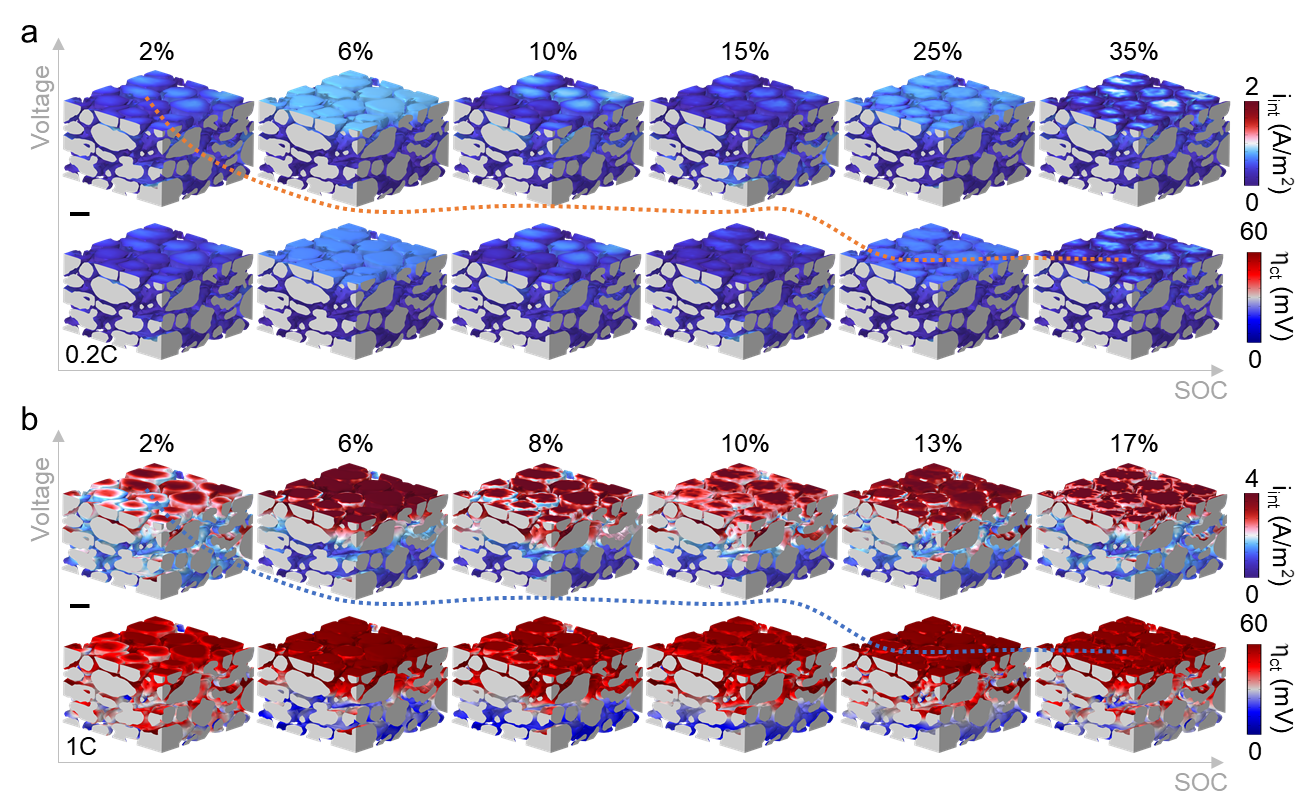


Figure S9. The phenomenon where the interfacial insertion current and potential within the electrode vary with equilibrium potential pulses. (a) 0.2C, (b) 1C.


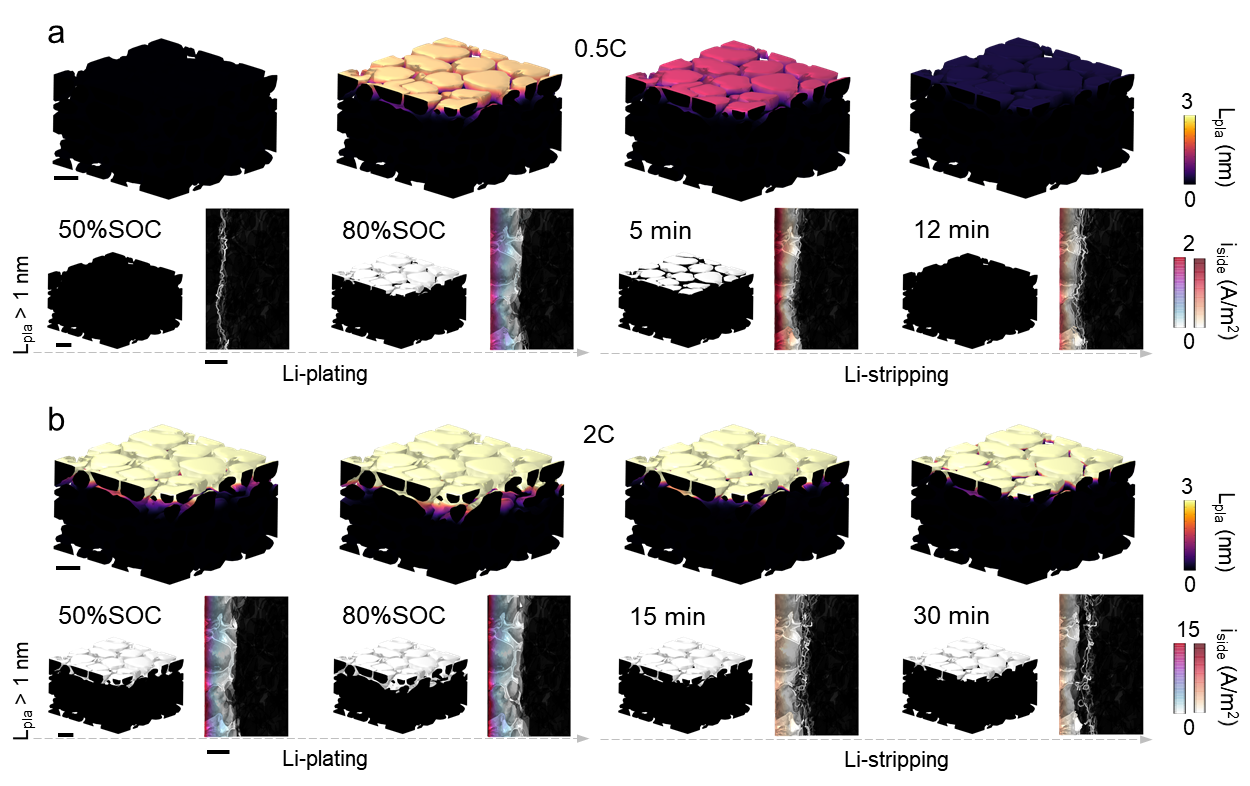


Figure S10. Lithium plating-stripping kinetics inside the electrode. (a) 0.5C, (b) 2C.


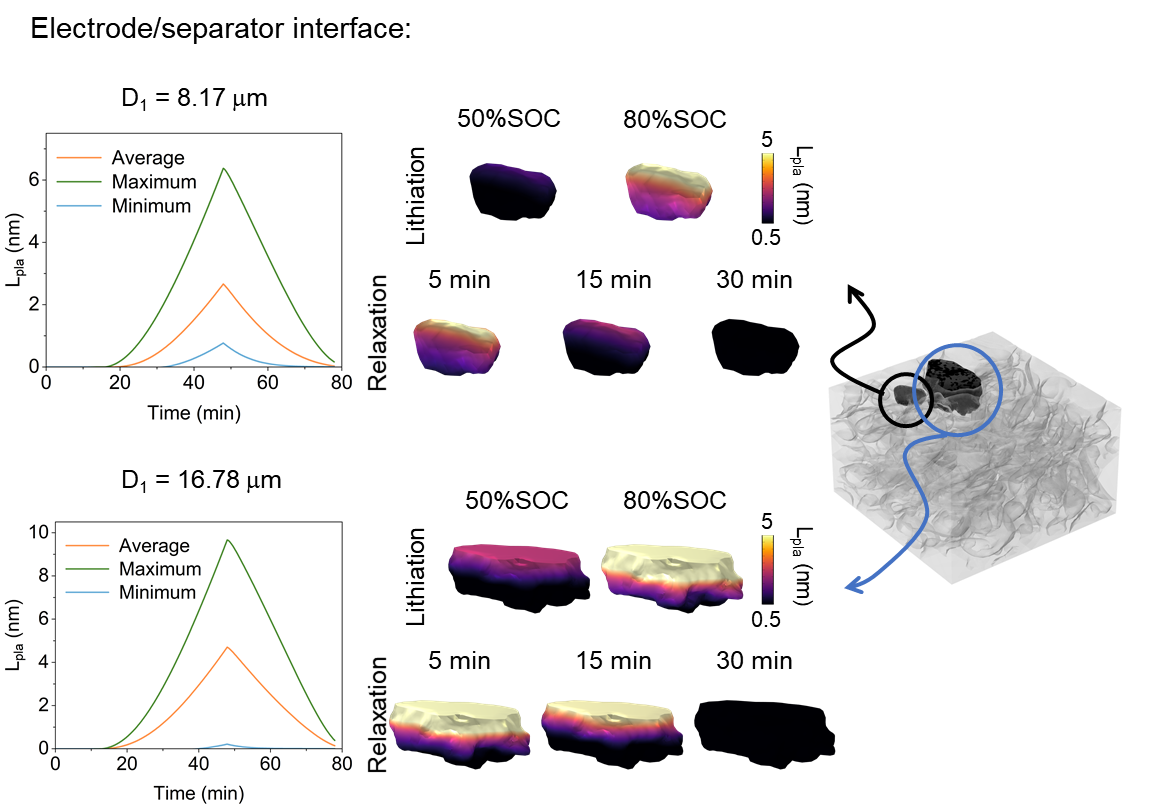


Figure S11. Effect of particle size on lithium plating deposition.


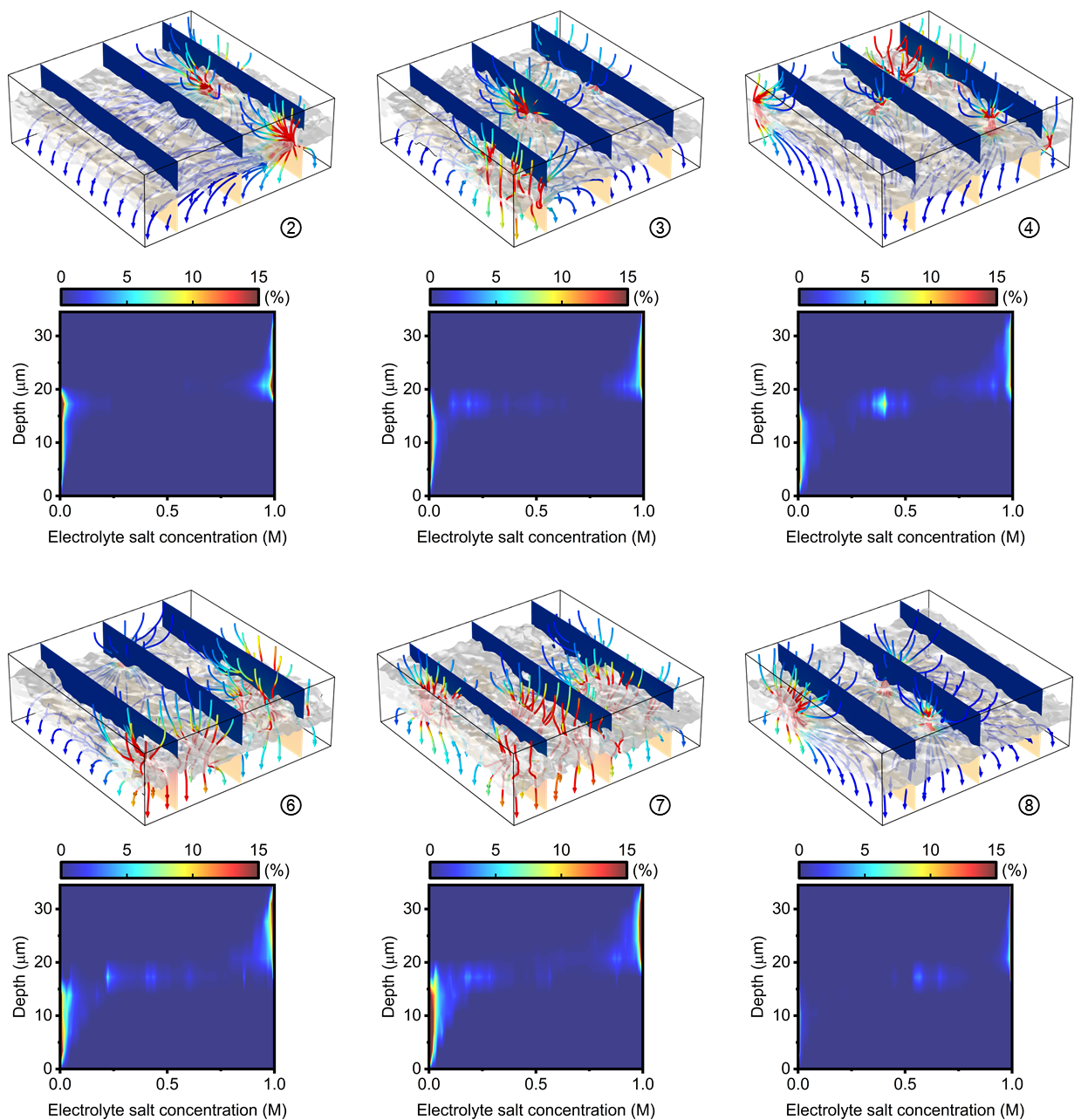


Figure S12. The effect of lithium plating regions on Li^+^ transport within the separator.


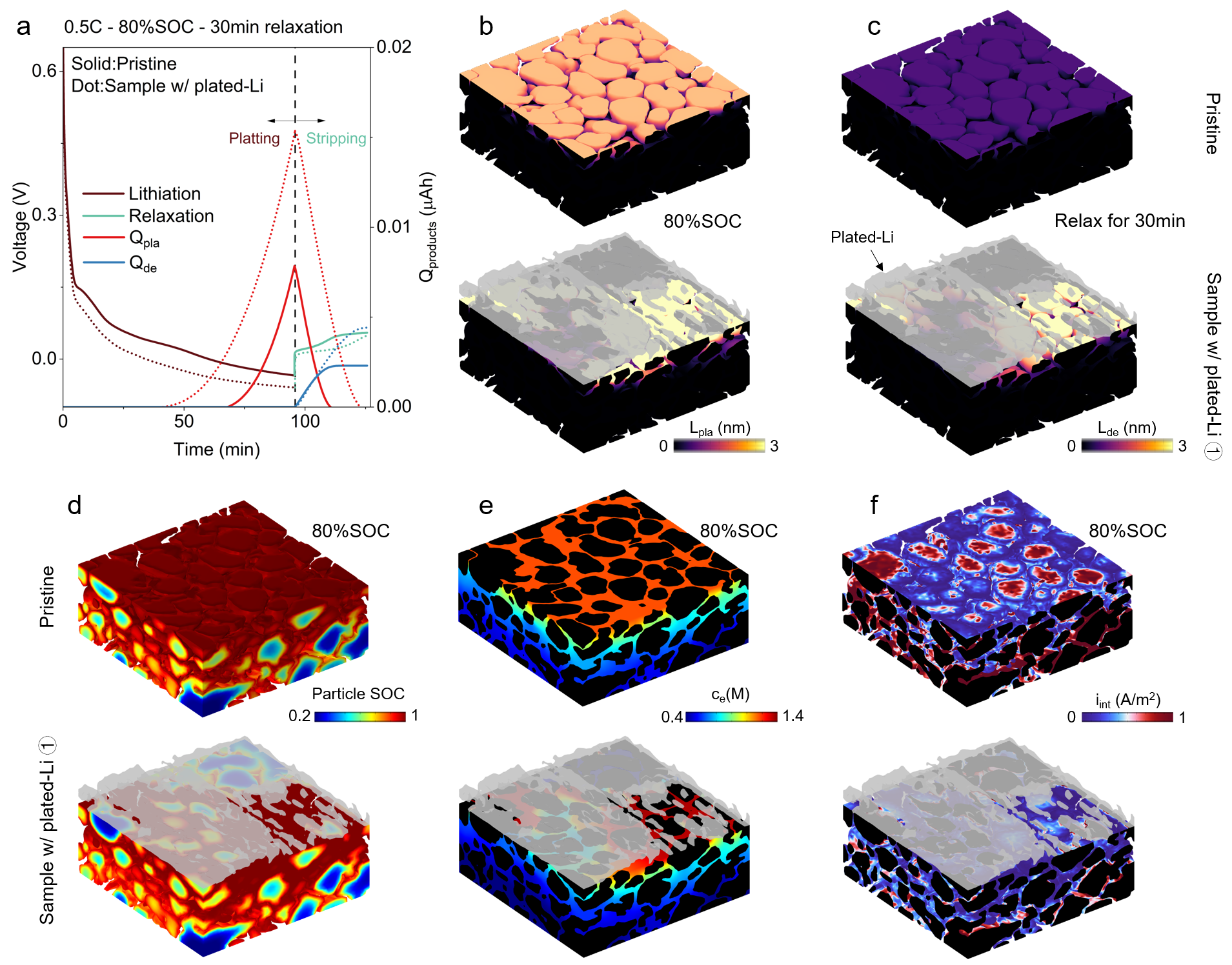


Figure S13. Effect of surface plated lithium on electrode discharge performance. (a) Discharge-relaxation curve at 0.5C. (b-f) Effects of electrode surface plated lithium on internal reactions and material distribution within the electrode. (b) Lithium deposition thickness, (c) dead lithium thickness, (d) particle SOC, (e) salt concentration in pores, (f) interfacial intercalation reaction current density.


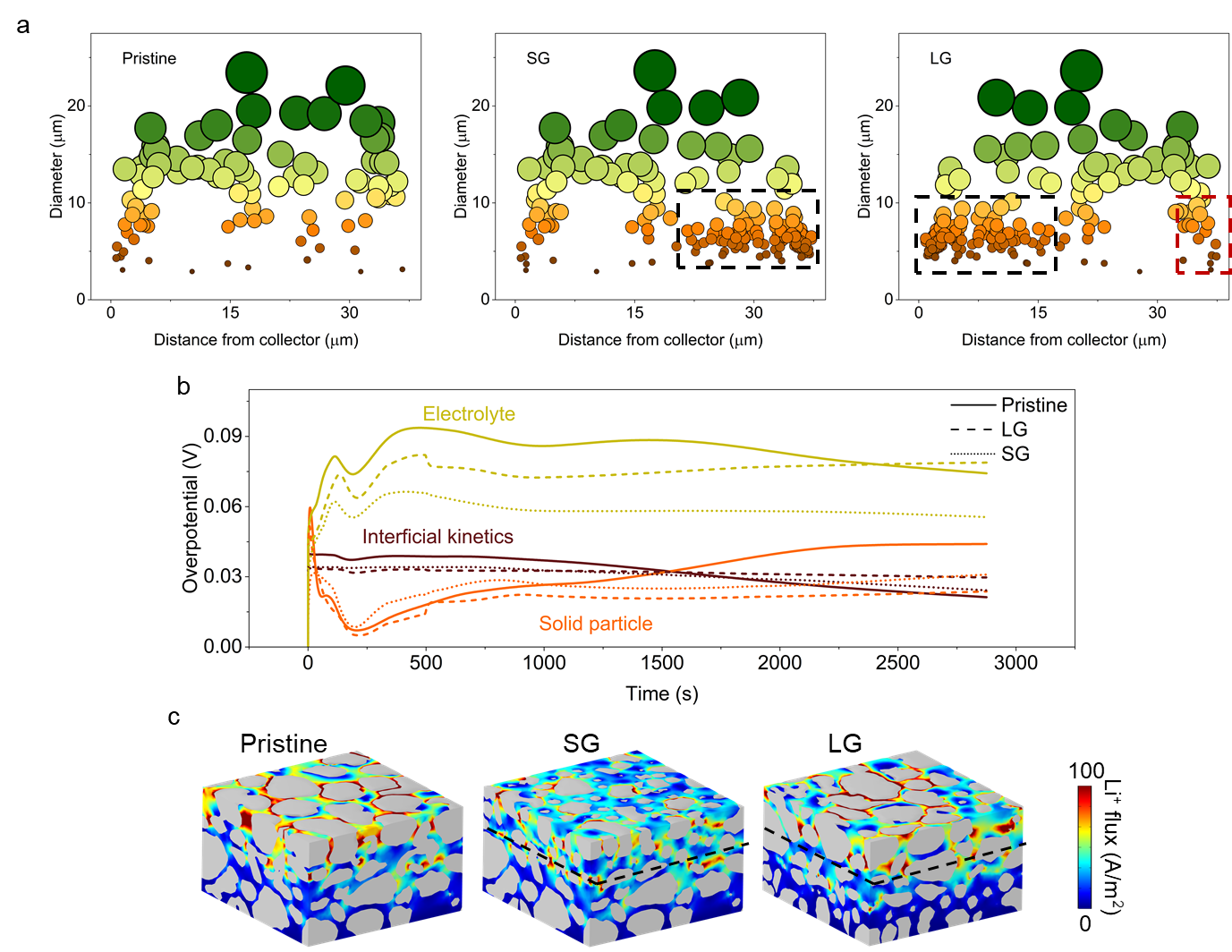


Figure S14. Physical and electrochemical properties of the modified electrode. (a) Particle size distribution in the thickness direction of the electrode. (b) The overpotential breakdown of the electrode. (c) Distribution of Li^+^ flux within the electrode at 6 min moment.


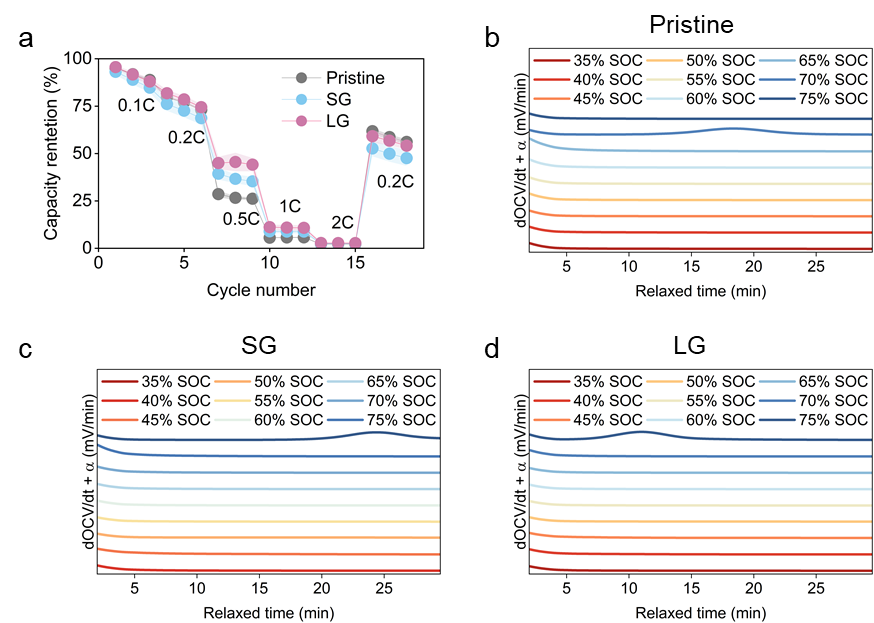


Figure S15. Rate performance and resistance to lithium plating of different electrodes. (a) Cyclic rate performance of electrodes. (b-d) Lithium plating onset SOC at 0.25C examined using differential voltage curves: (b) Pristine electrode, (c) SG electrode, (d) LG electrode.


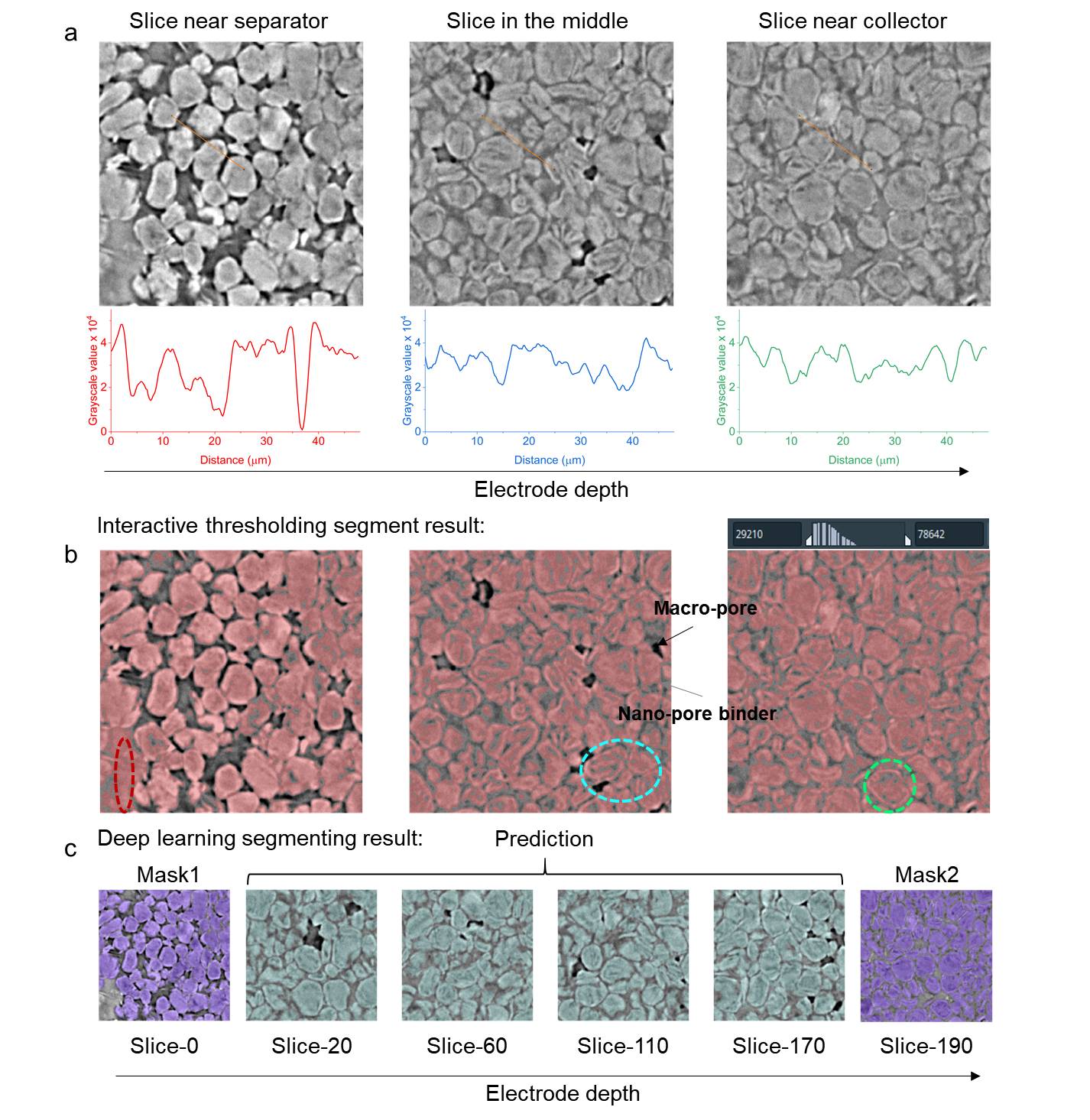


Figure S16. Deep learning methods for segmenting phase boundaries in graphite electrode XCT images. (a) Differences in CT image gray values at varying electrode thicknesses. (b) Failure of threshold segmentation based on gray-level histograms. (c) Deep learning-predicted segmentation results. In cross-sections at different electrode locations, the contrast between phases within the image exhibits significant variation due to X-ray attenuation rates, complicating manual segmentation. Due to the different gray-scale thresholds at different positions, this leads to over-segmentation and under-segmentation occurring locally within the same gray-scale range, as shown in (b). By labeling images at different positions and training a model using a deep learning network, good segmentation results are achieved at all positions across the electrode thickness.


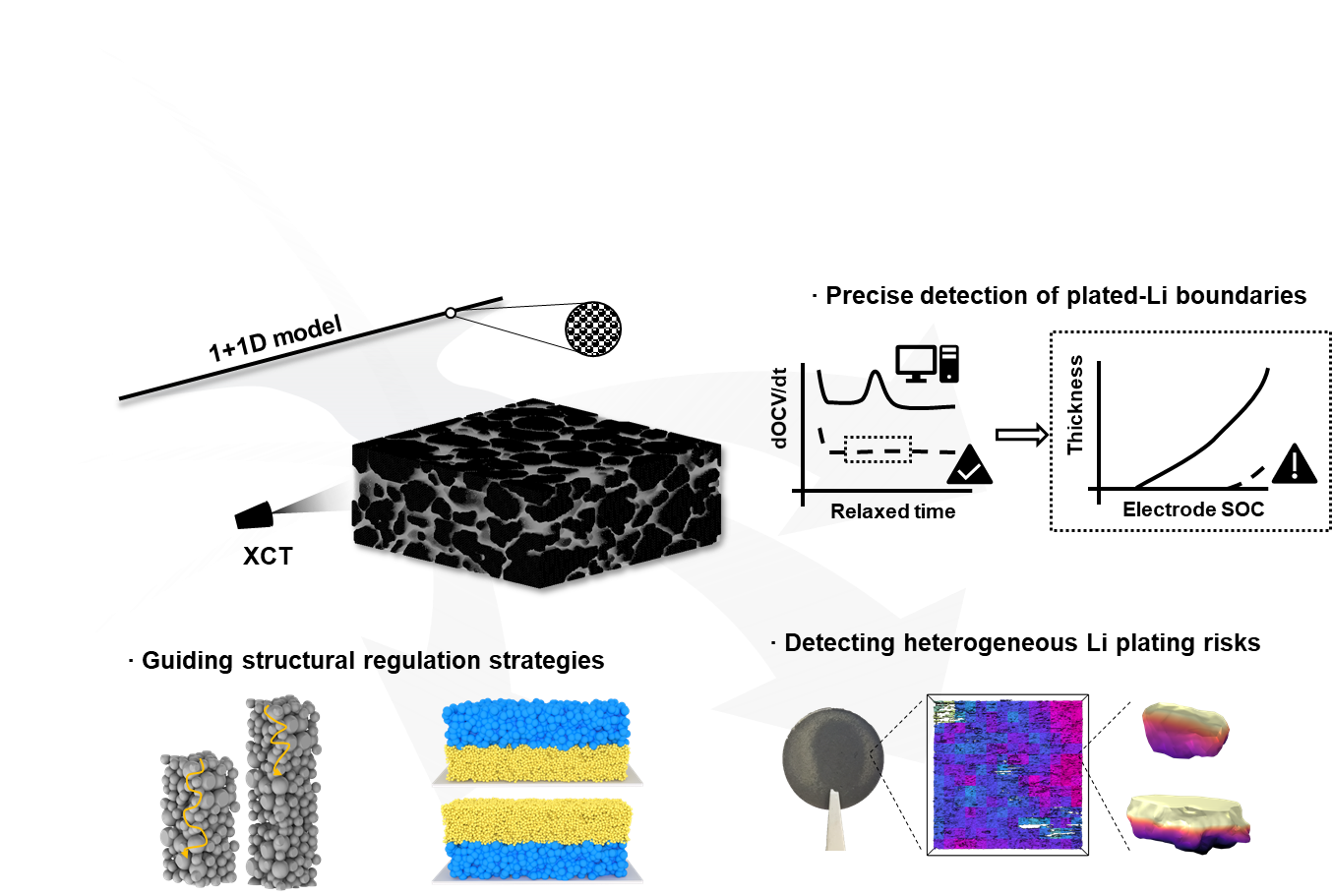


Figure S17 Workflow Diagram.

**Supplementary Method 1. Li plating-stripping reaction-sensing electrochemical model**

For the lithium plating-stripping reaction within the battery, a preliminary 1+1D model is established. The model domain includes a macroscopically homogeneous electrode region (particles + pores + CBD) and an additional particle domain. Key physical dimension parameters of the electrode region (electrode thickness, particle size) are estimated from SEM images (Figure 2).

The electrochemical model of the cell consists of equations describing electron (e^-^) conduction, ion (Li^+^) migration and diffusion, solid-state lithium (Li) diffusion, and charge transfer reactions [1]. Besides the established lithium plating-stripping reaction (Section 2.2.1), the insertion/deintercalation reaction at particle interfaces and Li^+^ diffusion/migration within the cell are described here. These partial differential equations (PDEs) and species conservation (e^-^, Li^+^, Li) operate in different abstract domains within the lumped region. The electron potential (ϕ_s_) in the active material and conductive domains is described by Ohm's law:

$\nabla\cdot J_{s}=0$ (S1)

(S2)

here, ϕ_s_ represents the electron current density, and $\sigma_{s}^{eff}$denotes the effective conductivity. Ion migration within the electrolyte generates a potential difference (ϕ_l_), which is likewise described by Ohm's law:

$\nabla\cdot J_{l}=0$ (S3)

(S4)

where J_l_ is the electrolyte current density, $\tilde{\varepsilon}$ is the total porosity, used only in the 1+1D model. τ is the pore tortuosity. κ_l_ is the electrolyte conductivity, and t_+_ is the cation mobility. The ion concentration (c_e_) in the electrolyte is described by the theory of dilute solutions:

$\widetilde{\varepsilon}\frac{\partial c_{e}}{\partial t}+\nabla\cdot J_{e}=0$ (S5)

$J_{e}=-\frac{\widetilde{\varepsilon}}{\tau}D_{e}\nabla c_{e}$ (S6)

where J_e_ denotes the ionic concentration flux, and D_e_ represents the diffusion coefficient of Li^+^ in the electrolyte. Diffusion of Li concentration within particles (c_p_) in the additional dimension is described by Fick's diffusion law:

$\frac{\partial c_{p}}{\partial t}+\nabla\cdot J_{p}=0$ (S7)

$J_{p}=-D_{p}\nabla c_{p}$ (S8)

in the equation, J_p_ represents the Li flux within the particle, and D_p_ denotes the diffusion coefficient of solid-phase Li, which is related to the lithium concentration at the graphite particle interface. The charge exchange reaction at the particle interface (i_int_) is described by the Butler-Volmer equation for symmetric cathode-anode reactions:

$i_{\mathrm{int}}=\alpha_{s}i_{0}\cdot\left[\exp\left(\frac{\alpha F\eta_{ct}}{RT}\right)-\exp\left(-\frac{\left(1-\alpha\right)F\eta_{ct}}{RT}\right)\right]$ (S9)

(S10)

where α_s_ is the specific surface area of the electrode, i_0_ is the exchange current density, α is the charge transfer coefficient, η_ct_ is the interfacial intercalation overpotential, and U_eq,Gr_ represents the equilibrium potential of the graphite electrode, which is a function of the interfacial lithium content. The interfacial overpotential relative to lithium metal (η_Li_) in the lithium plating-stripping reaction also includes the film resistance potential term formed by the SEI film and lithium metal [2]:

(S11)

$U_{film}=i_{tot}R_{film}$ (S12)

$R_{film}=\frac{\delta_{0}+\Delta\delta}{\sigma_{film}}$ (S13)

R_film_ represents the membrane resistance formed by the SEI film and the plated lithium deposit. δ_0_ denotes the initial film thickness, Δδ represents the change in film thickness, and σ_film_ indicates the film's conductivity. Since a single discharge cycle is considered, the change in secondary SEI film thickness caused by a small amount of lithium metal (c_Li_) can be neglected. Therefore, only the change in lithium plating thickness is accounted for in the thickness variation:

$\Delta\delta=\frac{\Delta c_{Li}M_{Li}}{a_{s}\rho_{Li}}$ (S14)

where M_Li_ is the molar mass of lithium metal, and ρ_Li_ is the density of lithium metal.

The PDE module in COMSOL Multiphysics software is used to model the kinetic physical processes involving lithium plating-stripping and deintercalation/intercalation. Applying current density at the external current collector boundary provides solid-phase electric flux. Based on Ohm's law, equivalent ionic flux is applied at the external separator boundary. Furthermore, the external separator boundary is defined as the reference potential. The total current density (i_tot_) at the electrode/electrolyte contact boundary drives ion/electron exchange, while solid-phase transport within the particles is solely driven by concentration gradients (i_int_). At the initial time, the graphite electrode is in a delithiated state, with an initial electrolyte concentration of 1 M and an initial SOC of 0.004. The equilibrium potential of the electrode is determined based on the discharge curve of the Li||Gr half-cell at 0.02C (Figure 4g). Detailed model parameters are listed in Table S2.


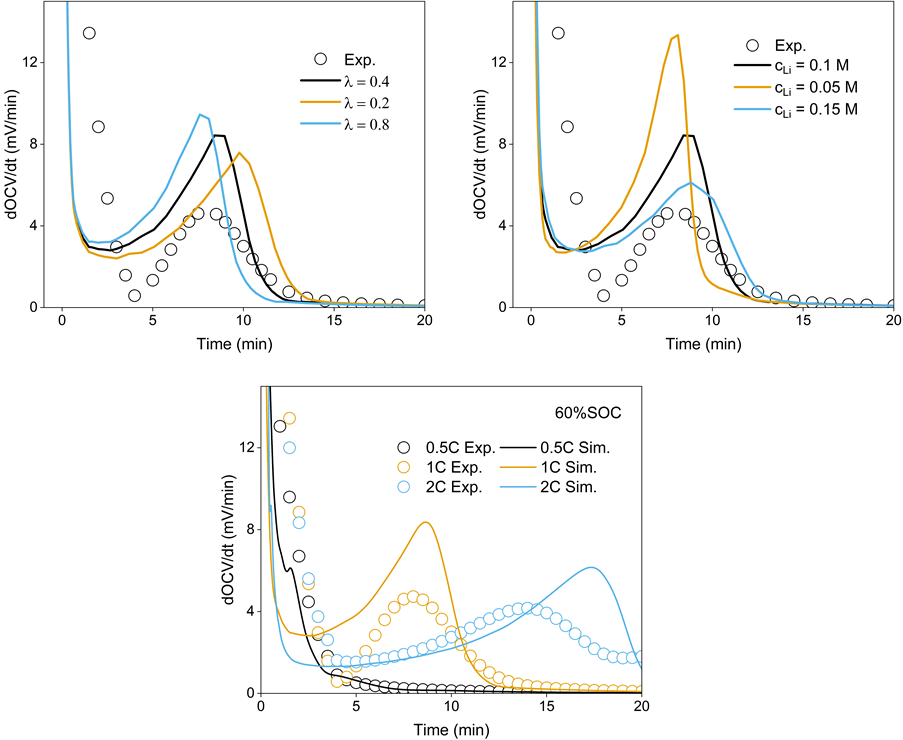


Figure S18. Correction of lithium plating-stripping model parameters. The irreversible coefficient of plated lithium and the reference concentration of lithium metal are crucial parameters determining the reversible efficiency of lithium metal in the lithium plating-stripping reaction. By fixing one parameter and adjusting the other, the model is calibrated to match the peak positions of the differential voltage curves measured experimentally. The calibrated parameters enable the model to closely approximate the experimental results for peak positions of the differential voltage curve across different discharge rates.

**Supplementary Method 2. Modeling setup for the effect of plated lithium on electrode performance**

The effects of plated lithium on the electrolyte transport and electrochemical performance within the electrode are simulated. First, the plated lithium is divided into nine sections identical in size to the graphite electrode. These plated lithium regions are then positioned at the same location above the electrode to eliminate the influence of relative positioning between the plated lithium and the electrode (Figure 5g). In the electrochemical model incorporating plated lithium, the electrode surface pores and active surface are blocked (Figure S19a). The blocking effect of plated lithium on Li^+^ transport in the separator direction is investigated using Dirichlet boundary conditions (Figure S19b-c). A concentration gradient is established in two opposite directions of the plated lithium layer to drive mass transfer:

$\frac{\partial}{\partial z}\left(D\frac{\partial c}{\partial z}\right)=0$(S15)

where, D is the diffusion coefficient for mass within the system, assumed to be 1 m^2^/s. The initial condition is defined as a 1 M ion concentration gradient along the thickness direction.


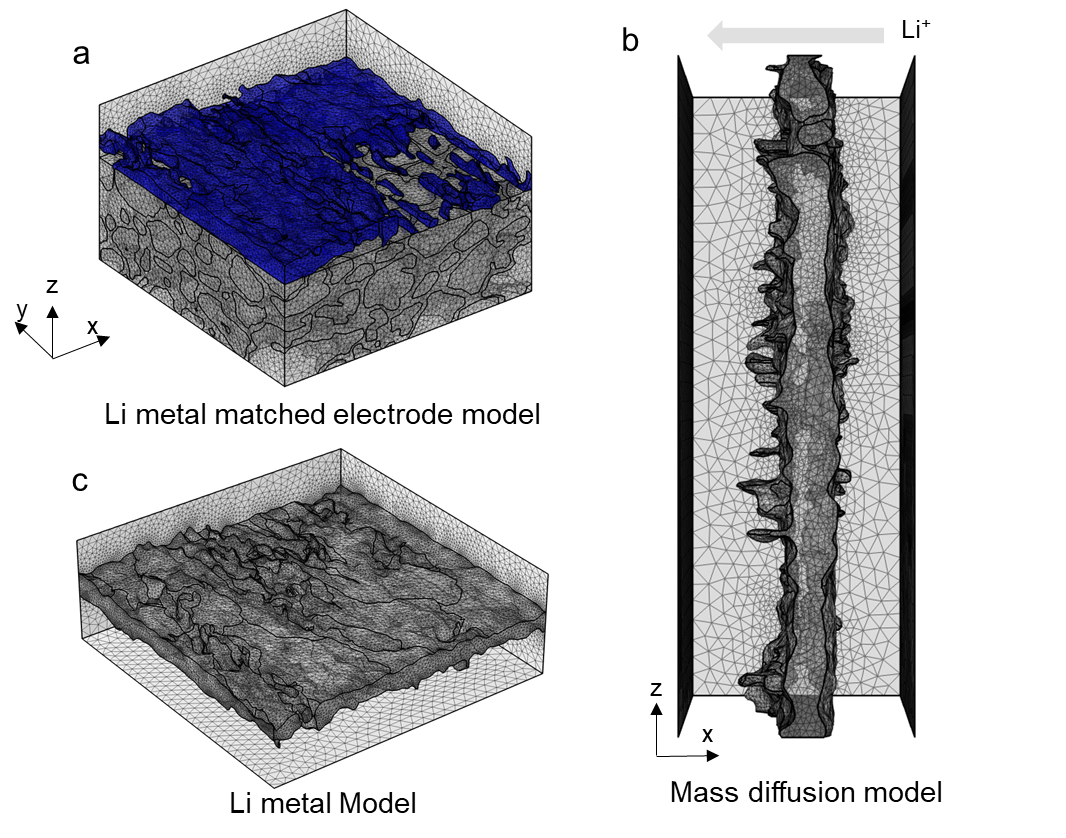


Figure S19. Numerical model simulating the effect of lithium plating on electrode performance. (a) Schematic diagram of the electrode model matching the lithium metal plating. (b) Schematic diagram of the mass diffusion model. (c) Model used to simulate the effect of lithium metal plating on electrolyte transport within the electrode.

**Supplementary Tables**

Table S1. Cell resistance at 50% SOC.

|  | R_0_ (Ω) | R_sei_ (Ω) | R_ct_ (Ω) |
| --- | --- | --- | --- |
| Pristine | 6.384 | 3.82 | 37.46 |
| SG | 4.954 | 1.462 | 21.26 |
| LG | 3.341 | 1.894 | 26.54 |
| 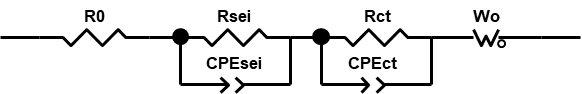 | | | |

Table S2. Electrochemical model parameters for lithium plating-stripping.

| **Model Parameters** | | |
| --- | --- | --- |
| **Electrode** | | |
| SOC_0_ | 0.004 | - |
| c_p,max_ (mol/m^3^) | 32643 | - |
| σ^s^_eff_ (S/m) | 50 | [3] |
| L_Gr_ (μm) ^**^ | 38 | Estimated based on SEM images (Figure 2c) |
| D_p_ (m^2^/s) | c_p_-dependent nonlinear function | [4] |
| U_eq,Gr_ (V) | Figure 4g | 0.02C Li\|\|Gr half-cell discharge voltage curve |
| α_s_ (1/m) ^**^ | 2.3×10^5^ | 3(1-$\check{\varepsilon}$)/r_p_ |
| r_p_ (μm) ^**^ | 8.5 | Estimated based on SEM images (Figure S7a) |
| $\check{\varepsilon}$ ^**^ | 0.34 | Estimated based on XCT images (Figure S7b) |
| **Electrolyte** | | |
| κ_l_ (S/m) | 10^-4^×c_e_(-10.35+0.074T-6.96×10^-5^T^2^+6.68×10^-4^c_e_-1.78×10^-5^c_e_T+2.8×10^-8^c_e_T^2^+4.94×10^-7^c_e_^2^-8.86×10^-10^c_e_^2^T)^2^ | [5] |
| D_e_ (m^2^/s) | 10^(-8.43-(54/(T-229-5×10^-3^c_e_))-2.2×10^-4^c_e_) | [5] |
| t_+_ | 0.38 | [6] |
| c_e,0_ (mol/m^3^) | 1000 | - |
| **Electrode/Electrolyte interface kinetics** | | |
| i_0_ (A/m^2^) | 1 | [7] |
| i_Li_ (A/m^2^) | 1 | [8] |
| c_Li_* (mol/m^3^) | 100 | Calibration based on dOCV/dt curves (Figure S17) |
| λ | 0.4 | Calibration based on dOCV/dt curves (Figure S17) |
| α | 0.5 | - |
| α_Li_ | 0.3 | [9] |
| δ_0_ (nm) | 10 | Calibration based on rate curves (Figure 2c) |
| **Physical parameters** | | |
| M_Li_ (kg/mol) | 6.94×10^-5^ | [2] |
| ρ_Li_ (kg/m^3^) | 534 | [2] |
| σ_film_ (S/m) | 5×10^-6^ | [10] |
| T (K) | 298.15 | - |
| F (C/mol) | 96485 | - |

^**^ marked parameters estimated in the 1+1D model.

Supplementary Note 1: The effects arising from the neglect of the CBD phase in graphite electrodes

The carbon binder domain exhibits low X-ray attenuation and is filled with nanopores, making this single phase extremely difficult to distinguish in grayscale images. Particularly in graphite electrodes, the graphite material itself is relatively insensitive to X-rays, rendering traditional full width at half maximum (FWHM) methods incapable of yielding satisfactory segmentation results (Figure S16). Consequently, segmentation of graphite electrode CT images has been largely omitted in nearly all relevant studies due to the inability to accurately locate their blurred boundaries. Moreover, the omission of the CBD phase appears to be an even greater consensus in related work [11, 12]. Therefore, in this study, to simplify the acquisition of graphite microstructure, we employ only advanced segmentation methods to distinguish between the graphite phase and the pore phase.

Additionally, due to their nanoscale dimensions, CBD phases readily adhere to particle surfaces. Supplementary high-magnification SEM images (Figure S20) reveal that the sparse distribution of CBD phases does not yet constitute any hindrance to mass transport or charge transfer at the electrode interface. To further validate this, we obtained estimates of the CBD phase structure (CBD volume fraction approximately 0.025) through simple calculations and virtual generation algorithms. Physical parameters were obtained using direct numerical simulation in Simpleware soft, yielding results with negligible variation (Figure S21).


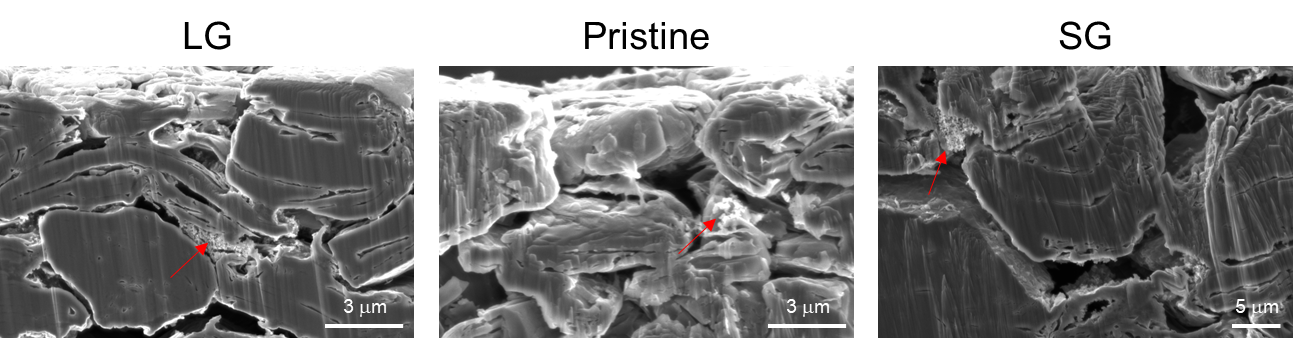


Figure S20 High-resolution SEM images of different electrodes.


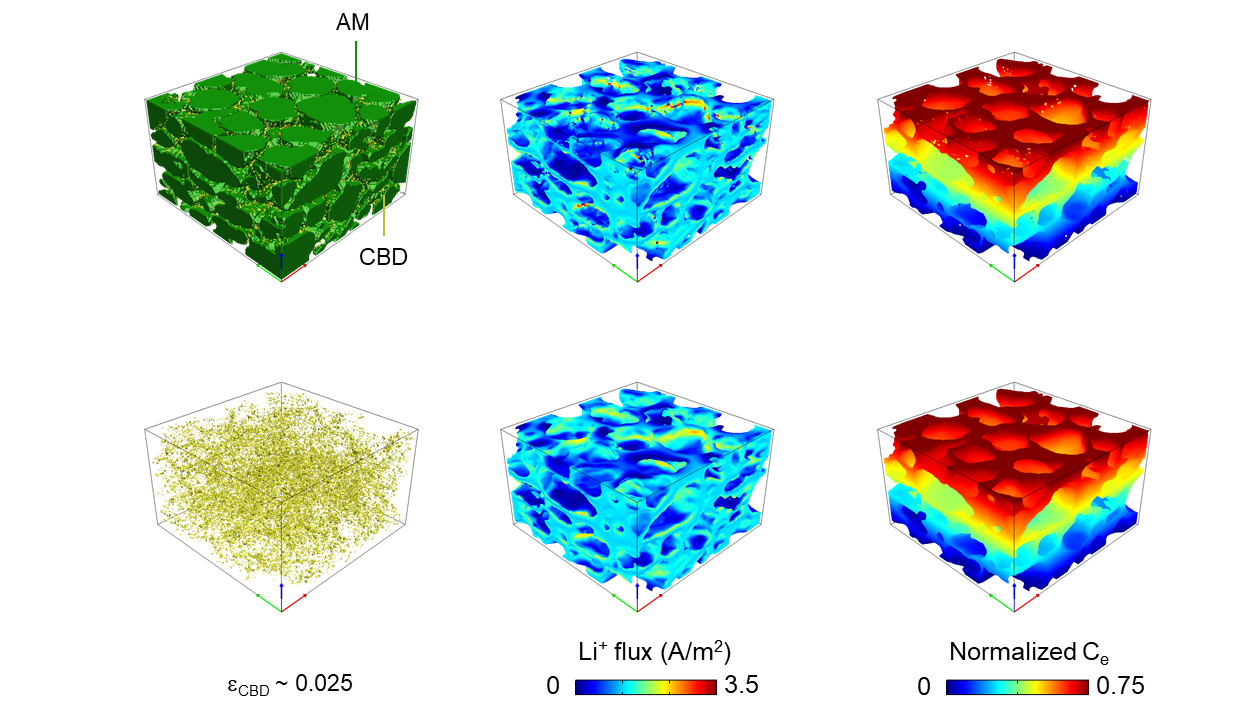


Figure S21 Virtual structures reveal the impact of CBD on ion transport.

Supplementary Note 2: Concentration distribution within particles in the solid solution model and the influence of equilibrium voltage

Galvanostatic intermittent titration (GITT) enables the identification of relaxation processes in electrode surface concentrations under open-circuit conditions, thereby yielding volume-averaged solid-state diffusion coefficients for the electrode. For phase-separated materials, GITT measurements on graphite electrodes in the rotary joint region yield extremely low solid-state diffusion coefficients. This leads to significantly different D_s_ values in the solid solution model, producing results resembling lithium-depleted and lithium-enriched phases. However, this creates catastrophic computational difficulties in the model's numerical calculations and necessitates extremely fine mesh refinement. In fact, such extremely low values stem from erroneous estimates of the effective active surface area at the particle scale, as revealed in the work of Angrawal et al. [13, 14]. Therefore, we employ a concentration-dependent solid-state diffusion coefficient parameter [15] in our solid solution model. This parameter is derived from density functional theory calculations of concentration-dependent tracer diffusion rates in graphite and has been utilized in numerous phase separation studies [16]. Although our simulations did not reproduce the concentration-step plateaus observed in phase-separated models, we employed this model to elucidate the microstructure-dependent nature of lithium plating side reactions. For this purpose, prior work has demonstrated that predictions from the solid solution model closely align with those from phase-separated models [3].

Additionally, when the open-circuit voltage undergoes a step change, it will exert a pulsed effect on the reaction rate within the electrode. This thermodynamically dependent phenomenon has been reported across numerous electrode materials. For instance, lithium iron phosphate exhibits more pronounced reaction heterogeneity compared to layered oxide cathodes, despite possessing a flatter open-circuit voltage plateau [17]. This occurs because steeper equilibrium potentials induce stronger reaction negative feedback, thereby effectively mitigating polarization heterogeneity [18].

Supplementary Note 3. Differences in simulation results between the 1+1D model and the microstructure model

The 3D microstructure model employs the same electrochemical parameter system as the 1+1D model. However, the 3D model is based on the actual structure of graphite electrodes, resulting in prediction outcomes distinct from those of the 1+1D model. Intuitively, the 3D microstructure model accounts for heterogeneous particle morphology, particle size distribution (Figure 4b-c), and heterogeneous pore distribution (Figure 4d-e). This results in more realistic heterogeneous electrochemical reactions and mass distribution within the electrode plane during operation. This consideration improves the model's agreement with experimental curves (Figure 4g and k), particularly for intercalation and lithium plating reaction kinetics.

However, due to differences in electrode specific surface area and particle size, the 3D microstructure model exhibits overestimated lithium plating rates and lower open-circuit voltages under identical lithium plating reaction parameters. This can be attributed to the greater heterogeneity of larger particles and smaller particle sizes (3D model: 11 μm, 1+1D model: 17 μm). At the same SOC, smaller particles exhibit greater lithium plating reaction intensity and higher lithium insertion capacity, leading to an earlier onset of the stripping plateau and a decrease in the voltage plateau.

Supplementary Note 4: Morphological changes of plated lithium during ex-situ CT scanning

Despite attempts to use epoxy resin adhesives or paraffin wax sealing, it was found that micron-sized bubbles and residual moisture in the medium could not be completely eliminated, leading to corrosion of the lithium plating layer over time. In a report on XCT of dendrites in solid-state lithium metal batteries, an 8-hour scan duration allowed sufficient oxidation of the lithium metal, yet the process results revealed no significant changes [19] . Furthermore, in situ TEM studies of the high-temperature oxidation process of lithium dendrites revealed that the outer membrane of lithium dendrites consists of a Li_2_O film composed of nanocrystals. After 5 hours at 160 °C, the film thickness was less than 60 nm [20]. Therefore, although the morphology of the plating lithium changes during oxidation, the alteration of the coating film can be considered negligible relative to the micrometer-scale plating thickness in this work.

Additionally, our scanning aims to observe the relative non-uniformity of lithium plating thickness, where the plating is fully exposed to humid air during oxidation to achieve uniform film thickening. This implies that the relative thickness of the coating across different regions should remain constant, and areas without lithium plating will not spontaneously develop it. From this perspective, the lithium coating exposed to air does not affect the analysis of the results from the uneven lithium plating reaction.

Due to limitations in liquid diffusion, lithium plating reactions occur exclusively on the electrode surface adjacent to the separator. This results in the distribution of plated lithium across the electrode surface, forming a layer of a certain thickness. Typically, the plated layer consists of interlaced dendritic structures forming block-like lithium, containing micrometer-scale pores, as shown in the SEM image in Figure 1f. However, lithium metal exhibits extreme insensitivity to X-rays, resulting in similar contrast to the pores (Figure 5c). Consequently, incorporating pores into the plated lithium region appears to be a common practice in related studies, as demonstrated in several synchrotron CT investigations [21, 22]. Therefore, in this work, micrometer-scale pores are considered integral components of the plated layer.

Supplementary Note 5: Microstructural models and experiments reveal the non-uniform lithium plating distribution in the full cell in a bottom-up manner.

The 3D structure of the lithium plating in Figure 5 was obtained by 3D-XCT scanning of the graphite electrode after disassembly of the coin half-cell. The non-uniform distribution of lithium plating thickness explains the uneven lithium plating reaction observed on the coin half-cell electrode (as shown in the inset of Figure 5a).

In the full cell, the separator homogenizes the Li^+^ flux from the cathode. Consequently, the phenomenon observed in the half-cell can readily be replicated in the full cell. Due to the larger surface area, the loading and thickness within the graphite electrode plane in the full cell are more likely to be non-uniform. This could exacerbate the uneven distribution of the lithium plating observed in the half-cell, potentially leading to more severe issues in the full cell.

Figure S13 further simulates the impact of dead lithium plating on subsequent lithium plating through an electrochemical model. Graphite electrodes with plating exhibit greater lithium plating reaction intensity at active surfaces (Figure S13b-c), indicating that the presence of dead lithium accelerates the non-uniformity of lithium plating reactions on the electrode surface. Additionally, Figure S11 illustrates the varying lithium plating reaction rates across different particle sizes, which similarly contributes to the uneven lithium plating layer on the electrode, as reported in the work [23]. Therefore, the conclusion regarding the uneven lithium plating reaction in the full cell is inferred based on both experimental results (Figure 5) and simulation results (Figures S11, S13), with this process depicted in Figure S22.


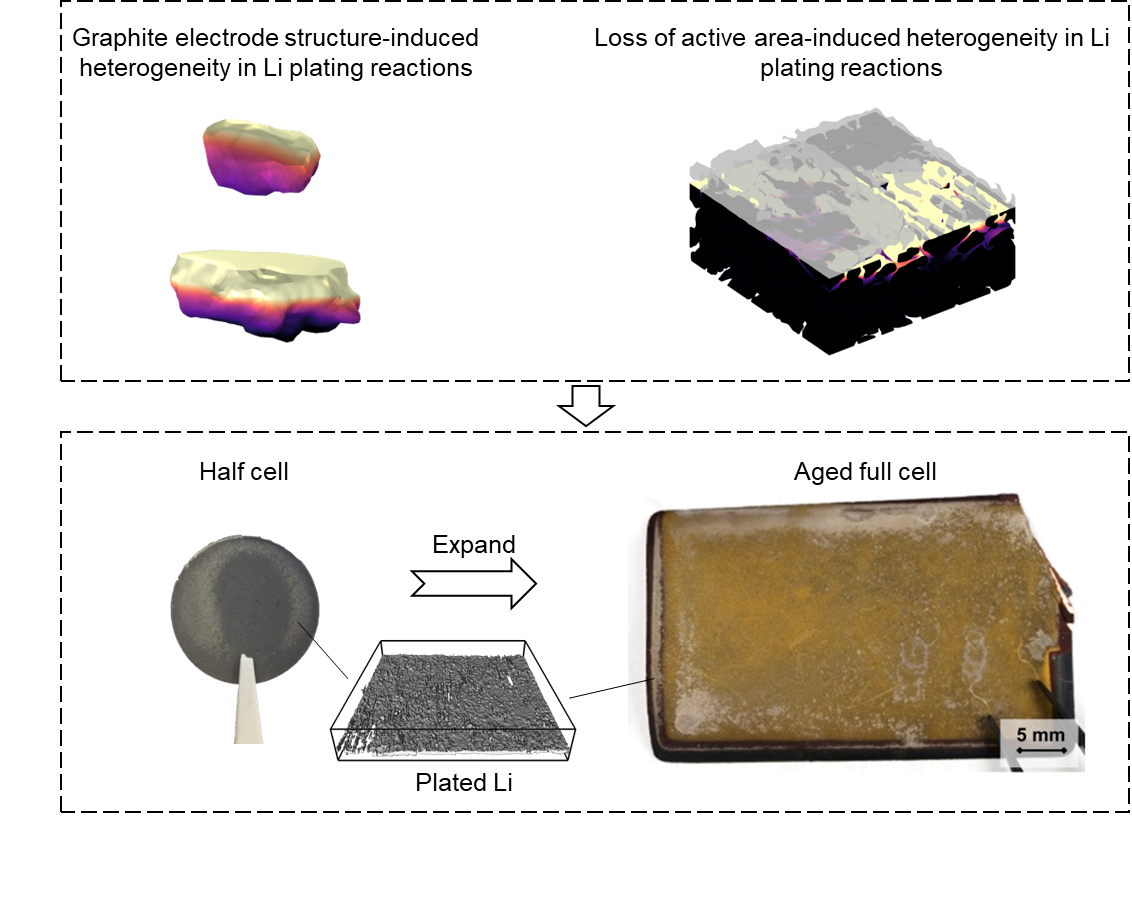


Figure S22 Simulation and experimental results reveal the non-uniform distribution of lithium plating on the graphite electrode of the full cell from the bottom up.

Supplementary Note 6: Simulation Results of Particle Size Control Strategy in Thicker Electrodes

Due to the liquid diffusion limitation effect on the 40μm electrode, the lithium plating reaction zone is confined to the separator side. As electrode thickness increases, the liquid diffusion limitation becomes more pronounced, further restricting the electrode reaction to a smaller area near the separator. Therefore, instead of using a fixed 40 μm thickness and its porosity structure parameters, we selected thicker and denser electrodes based on the same dominant kinetic mechanism of liquid diffusion limitation.

To verify that thicker electrodes yield the same conclusions as thinner ones, we thickened the electrodes using the mirroring operation in Simpleware software. The modified electrodes were processed by mirroring a local region of the initial electrode. Simulation results in Figure S23 show that when the thickness increases to approximately 80 μm, the relative voltage relationships among the three electrodes align with the voltage trends observed for the 40 μm electrode. However, the LG electrode exhibits lower overpotentials, while the improvement in overpotential for the SG electrode begins to diminish. Observing the onset of lithium plating, the thicker LG electrode delayed the initiation time and reduced the amount of lithium deposited. This simulation result broadly aligns with the experimental findings in Figure 7f. The engineered electrodes exhibit relatively stronger resistance to lithium plating, with the LG electrode significantly postponing the onset of lithium plating.


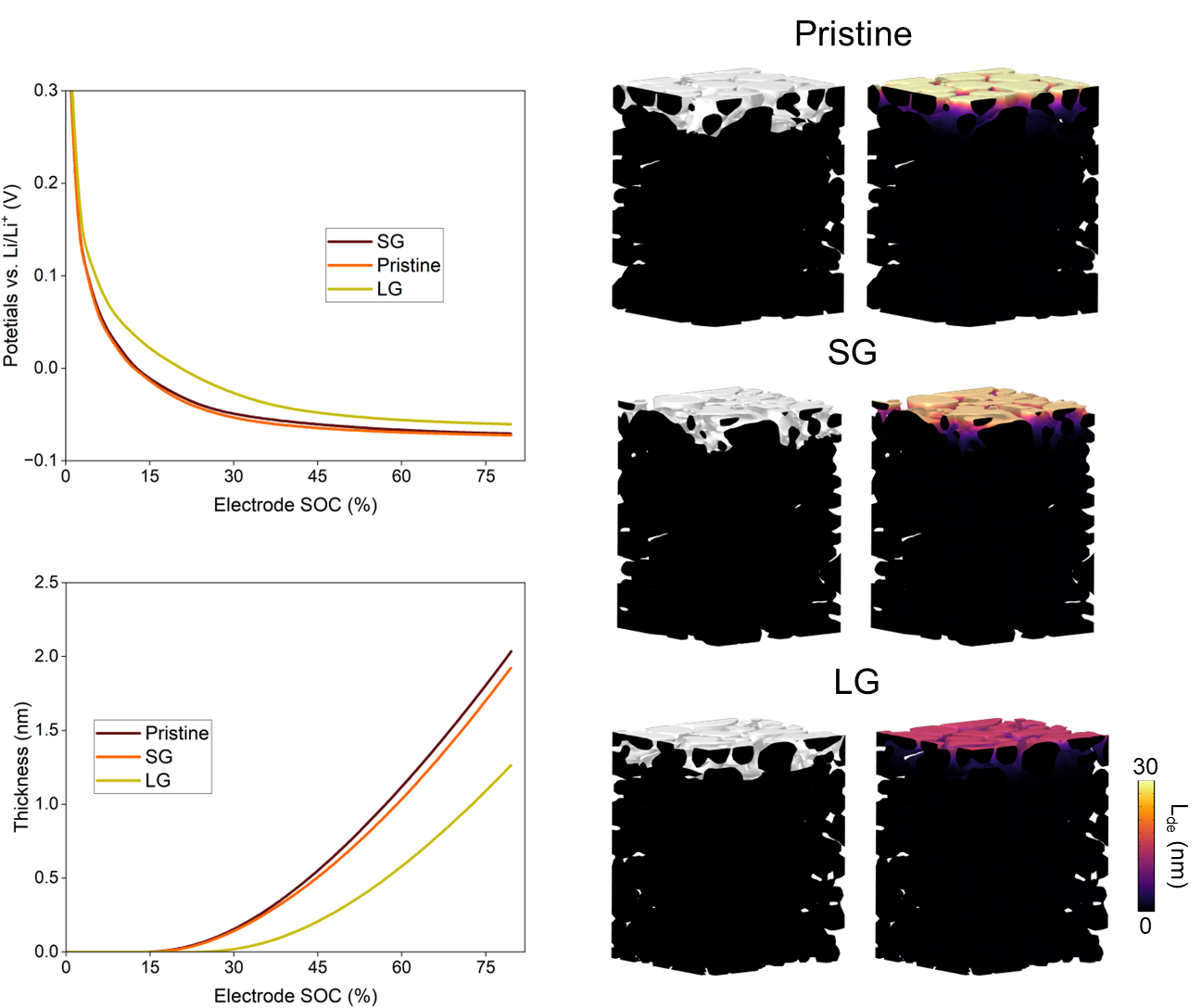


Figure S23 The effect of particle size control strategies on lithium plating reactions at greater thicknesses.

Supplementary Note 7: Experimental examination of thickness variations in electrodes with different particle size distributions

Several steps in electrode fabrication introduce errors, leading to non-uniform distribution of electrode thickness and mass. To address this, we strictly control mass and thickness uniformity. After rolling and secondary drying, electrode sheets undergo mass and thickness screening, requiring mass and thickness variations to be within 0.5 mg and 3 μm, respectively (Figure S24). The electrodes are then left to rest in a glove box for one day to fully release internal stresses. Subsequently, the samples are transferred for SEM scanning. The results in Figure 7b show that despite differences in the imaging angle of the electrode cross-sections, the thickness variations among the three electrodes become negligible, falling within approximately 5 μm. Within such a small difference in thickness, changes in porosity and current rate can be considered to have no effect on the results.


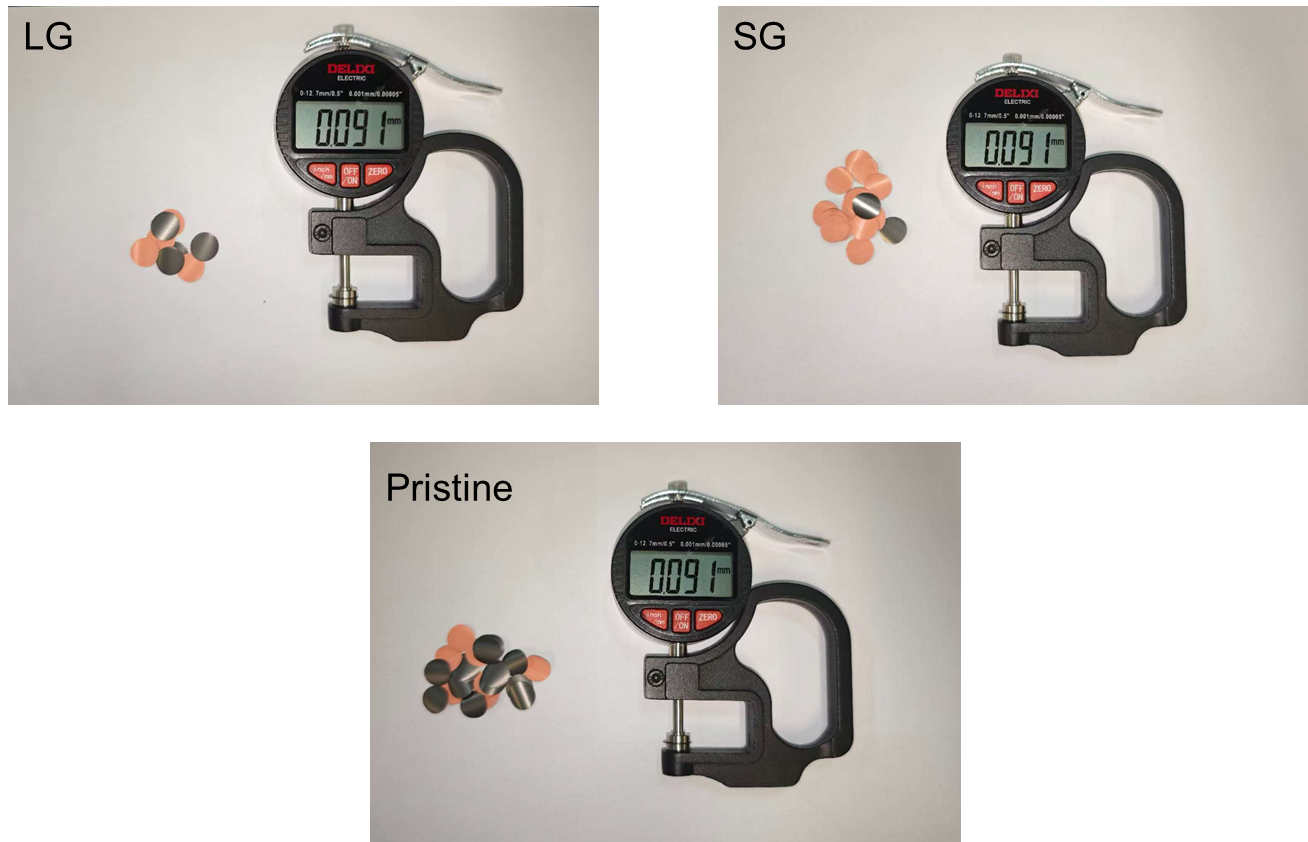


Figure S24 Screening different electrodes based on mass and thickness

Supplementary Note 8: SEI film-coupled models and experiments reveal the negligible impact of the SEI film on electrode performance.

Figure 7d results are primarily used to compare the SEI film, interfacial kinetics, and contact resistance among different electrodes. Due to the formation cycle and the use of a matched electrolyte, the SEI film resistance on the electrodes is very low. This is evident from the high-frequency portion of the EIS impedance spectra (Figure S25) and the fitting results in Table S1, where the SEI film resistance of each electrode is relatively low compared to the charge transfer resistance. This results in an initial SEI film resistance of approximately 10 nm as obtained from the model fitting (Table S1). This finding is supported by the second EIS test (Figure S25).

The SEI film formed by the reaction of deposited lithium with electrolyte solvents typically reduces battery lifespan. Since the work focused solely on the first plating process after battery formation, it was assumed that SEI film formation was minimal. This neglect is supported by the findings of Duan et al. [24] Their simulation of a 3.35 Ah full cell indicated that SEI membrane resistance loss accounted for approximately 0.3% of capacity retention after 80 cycles, while dead lithium formation caused about 2.4% capacity loss. Consequently, the SEI effect can be considered negligible in the simulation.

Although we employed identical SEI film thickness fitting conditions across different electrodes, particle size may also induce variations in the SEI film. To address this, we coupled the SEI model with a 1+1D model (Figure S26a) to examine the impact of the SEI film on electrode performance under diverse conditions, such as discharge rate, particle size, and electrode thickness. First, we fitted an approximate SEI film thickness (10 nm) by adjusting kinetic parameters related to SEI film formation, as shown in Figure S26b-c. Subsequently, we subtracted the results from the coupled SEI model from those of the original model under different conditions to isolate the impact of the SEI model. The results indicate that the SEI film has a negligible effect on electrode performance. Under thin electrode conditions in this work, the SEI film thickness remained nearly consistent across different particle sizes, resulting in a variation in deposited lithium thickness not exceeding 1 nm (Figure S26e). The SEI film induced a film potential variation of approximately 1 mV, reflected in a minor shift in electrode voltage (Figure S26d). For thicker electrodes, the current required to form the SEI film was weaker, and the variation in deposited lithium thickness remained below 1 nm, resulting in an interface potential drop of about 0.5 mV (Figure S26g). This indicates that increasing electrode thickness during operation actually mitigates the impact of the SEI film. At higher rates (2C), the increased current density resulted in a larger potential drop across the film, showing a voltage difference exceeding 6 mV (Figure S26i), but with minimal impact on the amount of deposited lithium. Based on the above changes in electrode material and electrochemical performance caused by the SEI film, we conclude that neglecting the SEI film does not affect the conclusions drawn from the work. This conclusion is supported by experiments, simulations, and related studies.


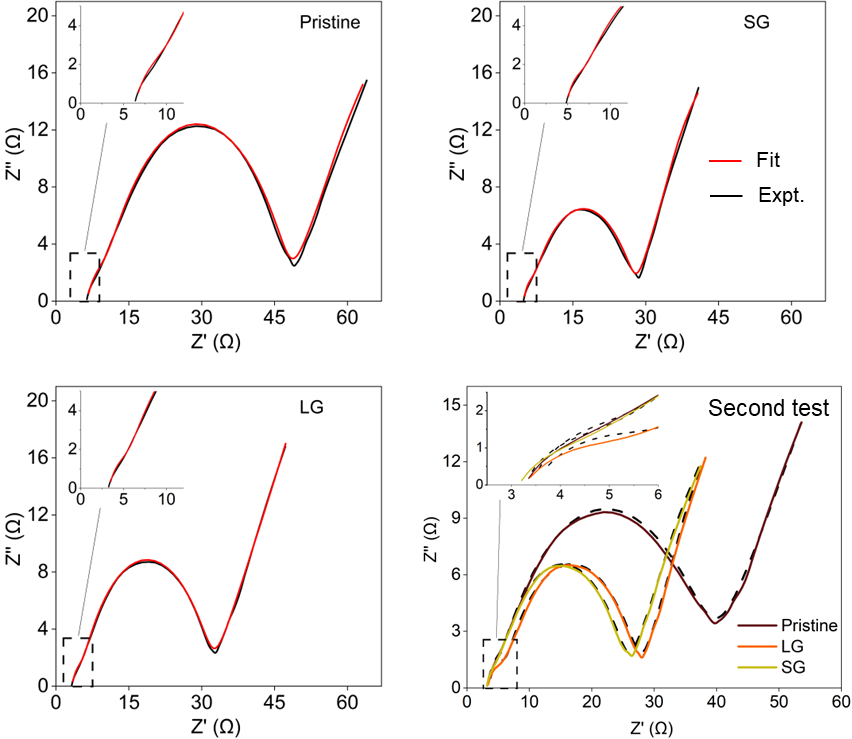


Figure S25 Focus on the SEI portion in the electrode EIS impedance spectrum and conduct a second EIS test.


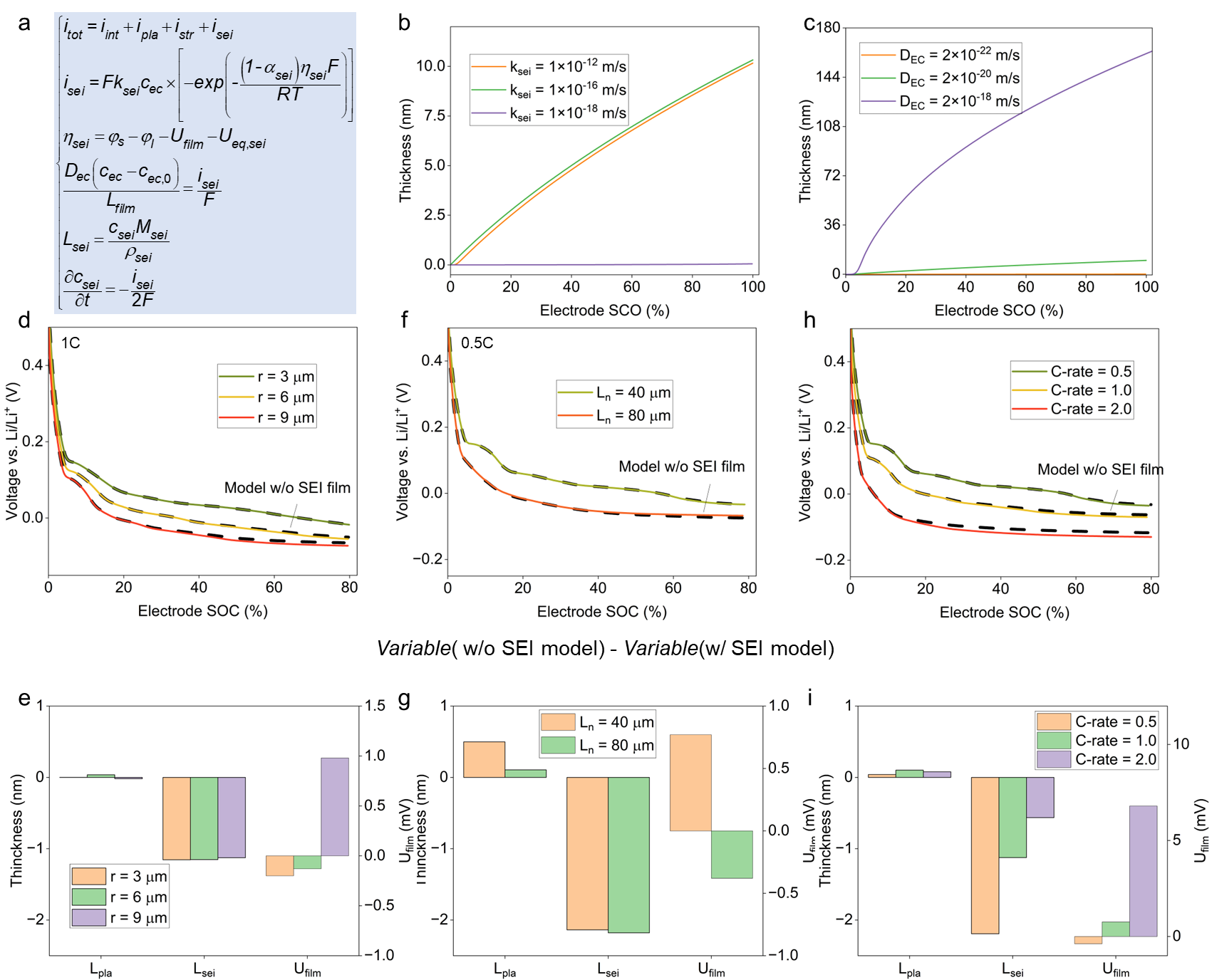


Figure S26 The SEI growth coupling model elucidates the influence of the SEI film under different structural conditions.

Supplementary Note 9: Representative analysis of graphite electrode volume and finite element mesh independence analysis

Through XCT scanning, we obtained grayscale images of the complete electrode, then employed deep learning for regional identification of graphite particle phases. Given the large volume range of the original image (196 × 272 × 38 μm^3^), we selected a localized 140 × 140 × 38 μm^3^ region for annotation to provide training targets for the deep learning algorithm (Figure S26a-b). After training, a 100 × 100 × 38 μm^3^ region was selected for microstructural characterization by comparing the original image with the training results. For finite element simulation, such large in-plane dimensions would necessitate an enormous number of mesh elements and pose significant meshing challenges. Therefore, a 64.5 × 64.5 × 38 μm^3^ region was extracted for meshing and subsequent simulations (Figure S26c). Thus, the in-plane dimensions for simulation were halved compared to the original segmentation. To validate volumetric representativeness, three additional distinct regions (Figure S26c) were compared by calculating porosity to assess the suitability of the selected volume. Results showed all regions exhibited similar porosity distributions, with the maximum standard deviation in in-plane porosity among the four regions remaining within 0.05.

For mesh generation, the particle phase and pore phase of the graphite electrode were first labeled. Subsequently, commercial software Simpleware, adapted for CT image meshing, was employed to perform the pre-FEM meshing steps. The minimum element size, surface mesh growth rate, and volume mesh growth rate are adjusted based on the characteristic dimensions of each phase, ensuring conformal nodes at boundaries. Linear free tetrahedral elements are meshed over the pore and particle phases using an adaptive algorithm.

To verify mesh independence, we simulated the case under 0.5C discharge using different minimum mesh cell sizes and compared capacity and average voltage across varying mesh counts. Figure S27 shows that as the grid size decreased from 2.8 μm to 1.2 μm, the number of grid cells increased by approximately 380000. This resulted in a 3.6% change in the predicted battery capacity at 0.5C and a 0.7% change in the average battery voltage. In this quantitative structural comparison, we did not precisely predict the onset of lithium plating. Therefore, such a small error can be considered within an acceptable range.


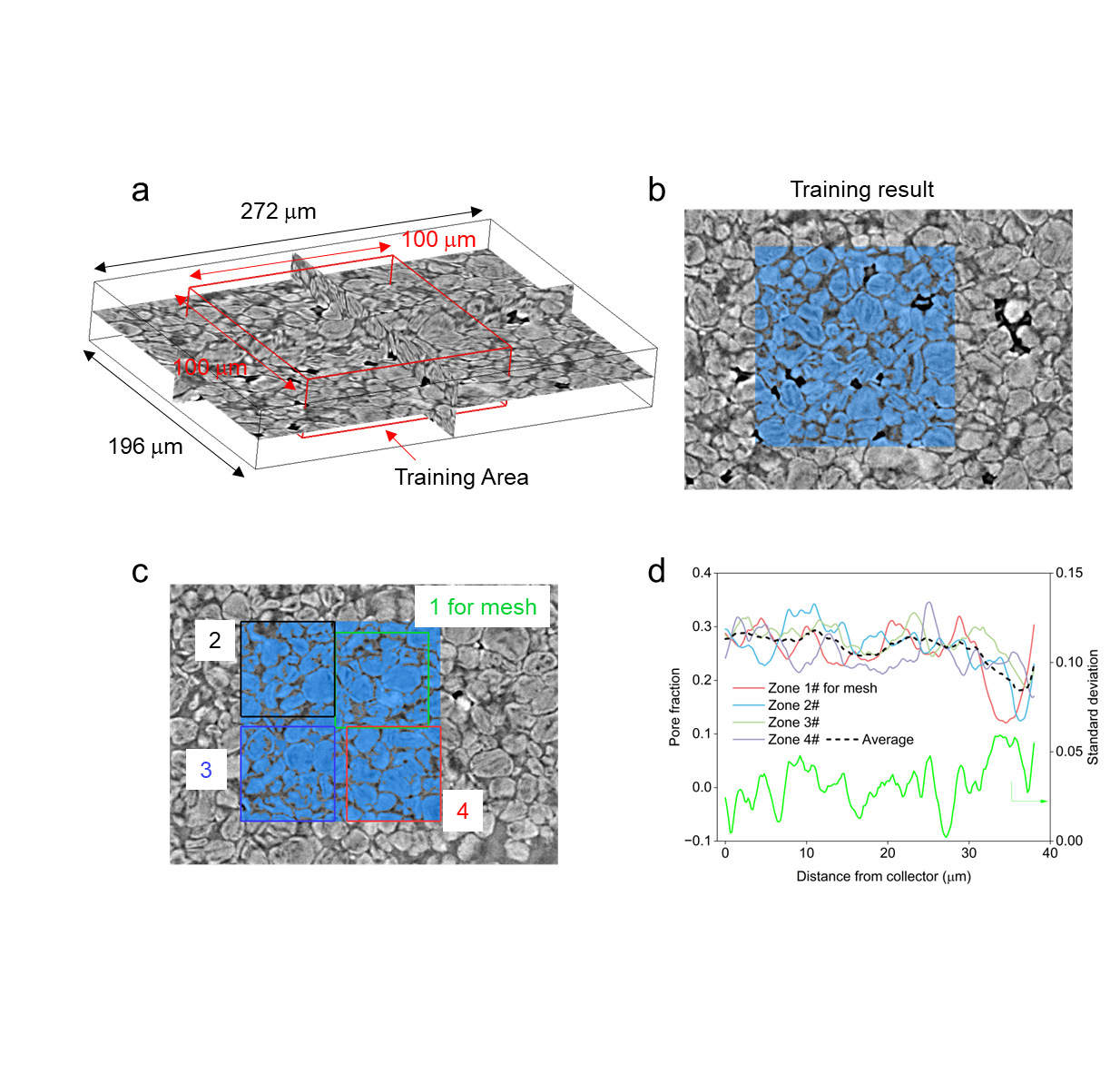


Figure S27 Representative analysis of electrode volume


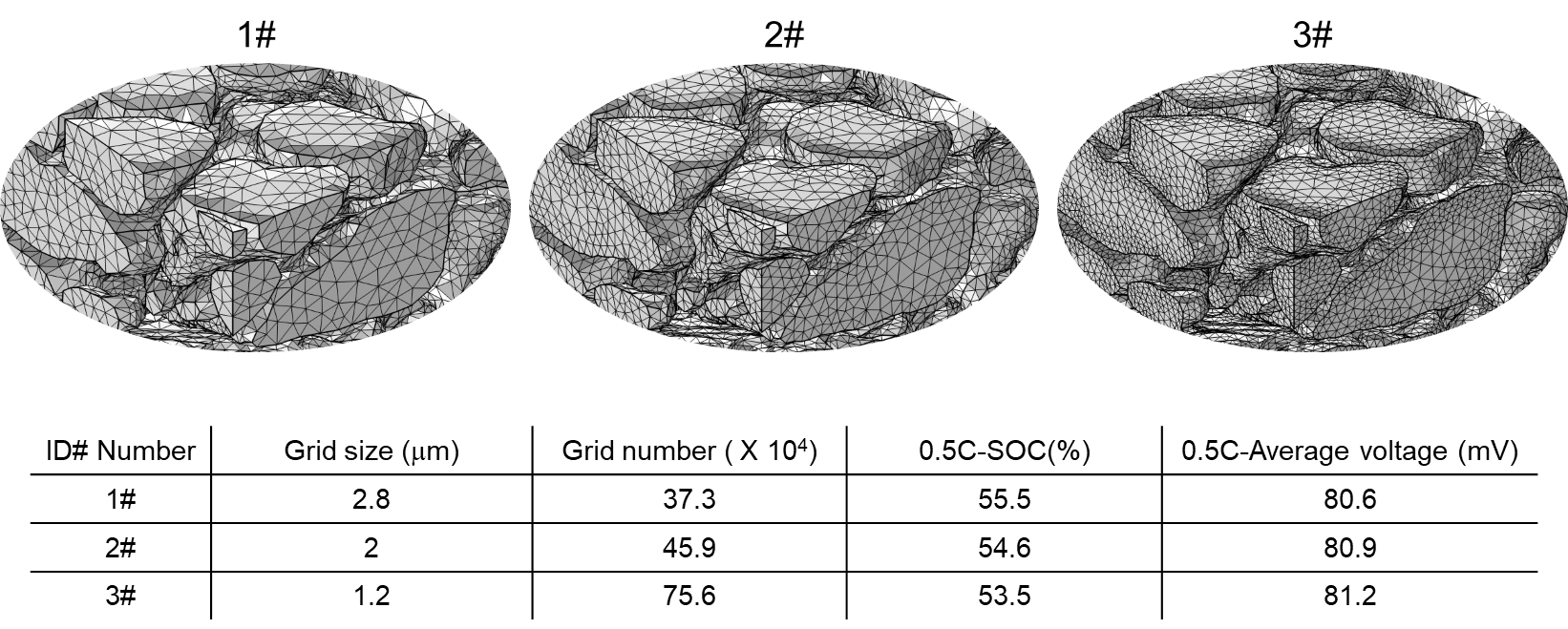


Figure S28 Grid independence verification

**References**

[1] Y. Ji, Y. Zhang, C.-Y. Wang, Li-Ion Cell Operation at Low Temperatures, Journal of The Electrochemical Society, 160 (2013) A636.

[2] X.-G. Yang, S. Ge, T. Liu, Y. Leng, C.-Y. Wang, A look into the voltage plateau signal for detection and quantification of lithium plating in lithium-ion cells, Journal of Power Sources, 395 (2018) 251-261.

[3] K.E. Thomas-Alyea, C. Jung, R.B. Smith, M.Z. Bazant, In situ observation and mathematical modeling of lithium distribution within graphite, Journal of The Electrochemical Society, 164 (2017) E3063.

[4] M. Kühne, F. Paolucci, J. Popovic, P.M. Ostrovsky, J. Maier, J.H. Smet, Ultrafast lithium diffusion in bilayer graphene, Nature Nanotechnology, 12 (2017) 895-900.

[5] L.O. Valøen, J.N. Reimers, Transport properties of LiPF6-based Li-ion battery electrolytes, Journal of The Electrochemical Society, 152 (2005) A882.

[6] L. Cai, R.E. White, Mathematical modeling of a lithium ion battery with thermal effects in COMSOL Inc. Multiphysics (MP) software, Journal of Power Sources, 196 (2011) 5985-5989.

[7] H. Chung, J. Kim, Y.S. Bae, J. Moon, Predictive modeling of lithium-ion battery degradation: Incorporating SEI layer growth and mechanical stress factors, Journal of Mechanical Science and Technology, 38 (2024) 6157-6167.

[8] J. Keil, A. Jossen, Electrochemical Modeling of Linear and Nonlinear Aging of Lithium-Ion Cells, Journal of The Electrochemical Society, 167 (2020) 110535.

[9] W. Mei, L. Zhang, J. Sun, Q. Wang, Experimental and numerical methods to investigate the overcharge caused lithium plating for lithium ion battery, Energy Storage Materials, 32 (2020) 91-104.

[10] X.-G. Yang, Y. Leng, G. Zhang, S. Ge, C.-Y. Wang, Modeling of lithium plating induced aging of lithium-ion batteries: Transition from linear to nonlinear aging, Journal of Power Sources, 360 (2017) 28-40.

[11] X. Lu, M. Lagnoni, A. Bertei, S. Das, R.E. Owen, Q. Li, K. O’Regan, A. Wade, D.P. Finegan, E. Kendrick, M.Z. Bazant, D.J.L. Brett, P.R. Shearing, Multiscale dynamics of charging and plating in graphite electrodes coupling operando microscopy and phase-field modelling, Nature Communications, 14 (2023) 5127.

[12] S. Scott, W. Du, R. Horwood, C. Lei, P. Shearing, A.P. Abbott, An Assessment of Blended Short Loop Recycled Graphite Electrodes Using X-Ray Micro-Computed Tomography, Advanced Energy Materials, 15 (2025) 2403498.

[13] S. Agrawal, P. Bai, Operando Electrochemical Kinetics in Particulate Porous Electrodes by Quantifying the Mesoscale Spatiotemporal Heterogeneities, Advanced Energy Materials, 11 (2021) 2003344.

[14] S. Agrawal, P. Bai, Dynamic interplay between phase transformation instabilities and reaction heterogeneities in particulate intercalation electrodes, Cell Reports Physical Science, 3 (2022) 100854.

[15] K. Persson, Y. Hinuma, Y.S. Meng, A. Van der Ven, G. Ceder, Thermodynamic and kinetic properties of the Li-graphite system from first-principles calculations, Physical Review B, 82 (2010) 125416.

[16] H. Lian, M.Z. Bazant, Modeling Lithium Plating Onset on Porous Graphite Electrodes Under Fast Charging with Hierarchical Multiphase Porous Electrode Theory, Journal of The Electrochemical Society, 171 (2024) 010526.

[17] Z. Li, F. Wang, Y. Gao, H. Wang, Z. Wang, Y. Yang, Q. Ai, M. Ge, Y. Liu, M. Meyer, T. Terlier, X. Xiao, W.-K. Lee, Y. Wang, J. Lou, A. Kiss, H. Agarwal, R. Stephens, M. Tang, Probing the Effect of Electrode Thermodynamics on Reaction Heterogeneity in Thick Battery Electrodes, Advanced Materials, 37 (2025) 2502299.

[18] H. Qu, M. Zhang, H. Ji, X. Wu, Y. Song, C. Chang, Z. Lao, Y. Song, J. Tang, Y. Zhu, G. Zhou, Thermodynamic Feedback Mechanisms for Mitigating Polarization in Lithium-Ion Batteries, Angewandte Chemie International Edition, 64 (2025) e202514404.

[19] C. Yildirim, F. Flatscher, S. Ganschow, A. Lassnig, C. Gammer, J. Todt, J. Keckes, D. Rettenwander, Understanding the origin of lithium dendrite branching in Li6.5La3Zr1.5Ta0.5O12 solid-state electrolyte via microscopy measurements, Nature Communications, 15 (2024) 8207.

[20] Y. Li, X. Li, J. Chen, C. Cai, W. Tu, J. Zhao, Y. Tang, L. Zhang, G. Zhou, J. Huang, In Situ TEM Studies of the Oxidation of Li Dendrites at High Temperatures, Advanced Functional Materials, 32 (2022) 2203233.

[21] H. Pan, T. Fu, G. Zan, R. Chen, C. Yao, Q. Li, P. Pianetta, K. Zhang, Y. Liu, X. Yu, H. Li, Fast Li Plating Behavior Probed by X-ray Computed Tomography, Nano Letters, 21 (2021) 5254-5261.

[22] L. Li, S. Basu, Y. Wang, Z. Chen, P. Hundekar, B. Wang, J. Shi, Y. Shi, S. Narayanan, N. Koratkar, Self-heating–induced healing of lithium dendrites, Science, 359 (2018) 1513-1516.

[23] H. Lin, Y. Hu, Y. Zhou, W. Hu, J. Lin, H. Luo, Y. Jin, D. Zhao, J. Chen, M. Tao, P. Shan, J. Liang, Y. Wei, Y. Yang, Unveiling the Onset, Evolution, and Kinetic Factors Associated with Lithium Plating on Graphite Electrodes in Lithium-ion Batteries, Advanced Energy Materials, 15 (2025) e02728.

[24] X. Duan, B. Li, J. Li, X. Gao, L. Wang, J. Xu, Quantitative understanding of lithium deposition‐stripping process on graphite anodes of lithium‐ion batteries, Advanced Energy Materials, 13 (2023) 2203767.
